# The political reference point: How geography shapes political identity

**DOI:** 10.1371/journal.pone.0171497

**Published:** 2017-02-16

**Authors:** Matthew Feinberg, Alexa M. Tullett, Zachary Mensch, William Hart, Sara Gottlieb

**Affiliations:** 1 Rotman School of Management, University of Toronto, Toronto, ON, Canada; 2 Department of Psychology, University of Alabama, Tuscaloosa, AL, United States of America; 3 Department of Psychology, University of California, Berkeley, CA, United States of America; Mälardalen University, SWEDEN

## Abstract

It is commonly assumed that how individuals identify on the political spectrum–whether liberal, conservative, or moderate–has a universal meaning when it comes to policy stances and voting behavior. But, does political identity mean the same thing from place to place? Using data collected from across the U.S. we find that even when people share the same political identity, those in “bluer” locations are more likely to support left-leaning policies and vote for Democratic candidates than those in “redder” locations. Because the meaning of political identity is inconsistent across locations, individuals who share the same political identity sometimes espouse opposing policy stances. Meanwhile, those with opposing identities sometimes endorse identical policy stances. Such findings suggest that researchers, campaigners, and pollsters must use caution when extrapolating policy preferences and voting behavior from political identity, and that animosity toward the other end of the political spectrum is sometimes misplaced.

## Introduction

When it comes to politics, how do you identify? Slightly conservative? Extremely liberal? Moderate, middle of the road? Academics, political campaigns, and pollsters commonly use people’s political identity as a heuristic for classifying and making judgments about what people believe, who they will vote for, and whether they should be targeted for political persuasion. The lay public often uses political identity to determine whether someone fits into their political ingroup or outgroup and is therefore deserving of respect or derogation [1]. Indeed, research suggests that although explicit prejudice is on the decline in most domains (e.g., race), it is on the rise when it comes to political identity [2, 3]. A core assumption made in each of these cases is that political identity has universal meaning; what one person understands to be a conservative (liberal) is what another person understands to be a conservative (liberal). But, do political identities have consistent meanings across people and places, or might researchers, campaigners, pollsters, and the lay public be judging a moving target (c.f., [4–9])?

Much research suggests that individuals arrive at their political identity through a confluence of bottom-up influences, including genetics [10, 11], physiology [12], personality [13], fundamental needs and motivations [14, 15], and moral values [16, 17]. Other research highlights the top-down influence of political elites and the media [9, 18]. Rarely looked at empirically is the possibility that horizontal influences–those exerted by the people around us–could be integral in how we understand and determine our own political identity. Abstract judgments about ourselves typically entail social comparison processes and a shared understanding of what it means to be part of a particular group [19–22]. Thus, choosing a political identity does not occur in a vacuum; instead it reflects what one’s social environment tacitly defines as liberalism and conservatism.

It stands to reason, then, that when people determine where they fall on the political ideology spectrum they will rely on perceptions of those around them. If so, where individuals live should have an important impact on how they identify because different locations diverge in how left or right leaning they tend to be overall, and thus should exert different social influences. For example, “moderately liberal” might mean something different to someone in a relatively Republican region of the country than it does to someone in a Democratic region. As such, we hypothesize that the political “blueness” or “redness” of people’s locations will influence where they view themselves on the political spectrum.

This reasoning is consistent with two competing hypotheses. First, it is possible that people feel pressure to conform to the identity that is prevalent in their location [23]. In other words, one might have to hold particularly liberal views to resist conformity and identify as a liberal in a red state, or particularly conservative views to identity as a conservative in a blue state. If this were the case, then location should “pull” people’s identities out of alignment with their views; as states get bluer, the same identity would be associated with more right-leaning views, and as states get redder, the same identity would be associated with more left-leaning views. We deem this first hypothesis the “identity conformity hypothesis”.

Alternatively, it is possible that people use the identity of those around them as a “political reference point”–a point with which to compare themselves and adjust accordingly [19]. In red locations, if individuals perceive their political stances to be to the left of most of those around them, they may identify as strongly liberal *even though* their positions (nationwide) are not nearly as extreme as those individuals identifying as strong liberals who live in blue locations. In contrast, individuals in blue locations who perceive their politics to be to the right of those around them will be more likely to identify as strongly conservative *even though* their positions (nationwide) are much more moderate than those identifying as strongly conservative in red locations. In other words, the political reference point in blue and red locations differs, and when individuals determine where they stand on the political spectrum, they will compare themselves to their respective reference points.

Both alternatives share an important implication: how a person identifies on the political ideology spectrum takes on different meaning depending on where people live. We test this possibility by measuring participants’ self-reported political identity and their attitudes on various policy issues, examining whether the relationship between political identity and policy attitudes shifts due to the blueness versus redness of one’s location. We start by exploring state level effects with data from the American National Election Study (ANES). Then, we examine whether the effects replicate at the more nuanced county level using data we collected specifically to test our hypotheses.

With data from the ANES (*N*_issues_ = 1,809, *N*_voting_ = 3,862) we assessed the correspondence between participants’ reported political identity (e.g., “slightly conservative”) and their stances on 9 political issues (e.g., abortion). We examined whether this correspondence shifts depending on state “blueness” (i.e., the percentage of voters that voted for Obama vs. Romney in the 2012 presidential election), testing specifically whether individuals–regardless of political identity–hold different policy positions depending on their state. We tested whether this effect would replicate in a second sample (*N*_issues_ = 1,269, *N*_voting_ = 1,056), allowing for a more refined test at the county level to see whether the effect generalized to a different set of 10 politically relevant issues. In both samples we also tested whether blueness (this time calculated from the previous election’s results to avoid redundancy) predicted voting in the upcoming presidential election.

## Materials and methods–Study 1

For extended methods and results for Study 1 and 2, see Supplementary Information available online. All research reported below was approved by the University of Toronto Research Ethics Board (#31102). Participants consented electronically by clicking an “I consent” button, a method approved by the ethics board.

In Study 1 we examined whether specific political identities (e.g., “strong conservative”) were associated with different positions on issues depending on one’s state of residence. To do so we used data from the publicly available American National Election Survey (ANES) dataset. Participants indicated their political identity using a 7-point scale (1 = Extremely liberal, 2 = Liberal, 3 = Slightly liberal, 4 = Moderate, middle of the road, 5 = Slightly conservative, 6 = Conservative, 7 = Extremely conservative). Responses were reverse coded so that higher scores indicated greater liberalism.

Participants’ views on 9 political issues (e.g., affirmative action) were used to create a composite score called “issue position” (α = .79; higher scores indicate more liberal positions). This yielded a sample of *N* = 1809 participants for analyses involving issues.

From each participant’s state of residence we were able to determine the percentage of people in that state that voted Democrat or Republican in the 2012 national election. Values for percentage-voting-Democrat and percentage-voting-Republican were highly correlated, *r*(1809) = -.99, *p* < .001, so we used percentage-voting-Democrat (state blueness) values throughout.

The ANES dataset provided us with the opportunity to assess whether political identities were associated with different voting behavior depending on one’s state of residence. Participants indicated the person they voted for (1 = *Democrat*, 2 = *Republican*) during the 2012 Presidential election. We recoded responses such that higher values corresponding to voting Democrat. For analyses involving voting behavior we used state blueness values from the previous election (i.e., 2008) to avoid redundancy between the state blueness predictor variable and the voting outcome variable. For analyses of voting intentions, we collapsed political identity into three categories (conservative, moderate, and liberal) as we were primarily interested in comparing identity-consistent and identity-inconsistent voting across states (N.B. treating political identity as a continuous variable does not qualitatively change the results of either study). Participants were included in analyses if they voted for a Democratic or Republican candidate and responded to the identity item. This yielded a sample of *N =* 3,862 participants for analyses involving voting.

## Results–Study 1

**Fig 1:**
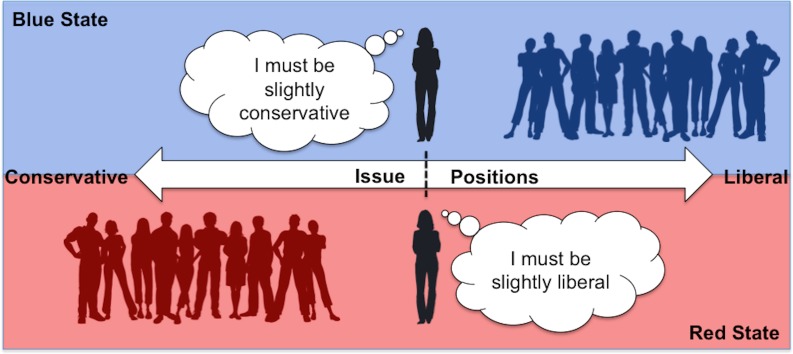
The political reference point.

The ANES data were consistent with the political reference point hypothesis (Fig 1) and not the identity conformity hypothesis. We ran a stepwise regression predicting issue position from political identity (centered; Step 1), state blueness (centered; Step 2) and their interaction (Step 3). This analysis revealed that even after accounting for differences in self-reported political identity, the bluer the state individuals lived in, the more their policy positions aligned with a liberal stance, *R*^*2*^_*change*_ = .004, *F*(1, 1806) = 12.32, *p* < .001 (Fig A in S1 File). Exploratory analyses revealed that there was also an unpredicted interaction between political identity and state blueness, *R*^*2*^_*change*_ = .002, *F*(1, 1805) = 8.11, *p* = .004. Simple slope analyses indicated that state blueness significantly predicted policy positions for both the conservatives and moderates, but not for liberals (Table B in S1 File). In other words, conservatives and moderates in blue states indicated more support for liberal policy positions than conservatives and moderates in red states, and the bluer the state was, the stronger their support was for liberal positions. This effect also extended to people’s actual behavior–people in bluer versus redder states were more likely to vote Democrat in the 2012 Presidential election, even after controlling for political identity, χ^2^(1) = 9.84, *p* = .002 (Fig B in S1 File). Thus, people who reported the same political identities differed in their political stances and voting behavior depending on the redness versus blueness of their state.

We also tested whether these effects replicated in 10 additional samples using ANES data from every election year going back to 1972 (*N*_issues_ = 8,336, *N*_voting_ = 12,842). Data collected over the past 44 years again demonstrated that even when accounting for political identity, the bluer the state individuals live in the more liberal the positions they will hold, *R*^*2*^_*change*_ = .005, *F*(1, 8333) = 57.15, *p* < .001, and the more likely they will be to vote Democrat, χ^2^(1) = 117.30, *p* < .001 (Figs C–X in S1 File; Tables E–BB in S1 File). In this case the interaction between political identity and state blueness was barely significant, *R*^*2*^_*change*_ = .000, *F*(1, 8332) = 3.99, *p* = .046, and exhibited a different pattern than that observed in the 2012 data (with the strongest effect of state blueness amongst moderates), suggesting that interactive effects may be inconsistent over time.

## Materials and methods–Study 2

In Study 2 we assessed the same question as in Study 1 with a sample collected specifically to test our hypotheses. Here, we were able to assess “blueness” at the county level, allowing a more nuanced test of our main question. We also did targeted recruiting of participants in order to ensure that the extremes of political identity (i.e., *strong conservatives* and *strong liberals*) were well-represented. Finally, this study also allowed us to test the generalizability of our previous results by testing different political issues.

Because there was little precedent for estimating these effects, we decided to collect a relatively large sample. For each political identity position (1 = *strong conservative*, 2 = *conservative*, 3 = *moderate conservative*, 4 = *moderate*, 5 = *moderate liberal*, 6 = *liberal*, 7 = *strong liberal*) we aimed to recruit 100 mTurk workers from red states and 100 from blue states from a large pre-screened sample (N of approximately 30,000). A total of 1,349 participants began the survey and 1,269 finished all relevant items. This sample of 1,269 people was used in analyses involving issue position (*M*_age_ = 39.80, *SD*_age_ = 12.94, 672 female; see Table D in S1 File for number of participants in each category).

Participants indicated their position (-*5 = strongly oppose*, *5 = strongly in favor*) on 10 political issues. Five issues were traditionally liberal (e.g., *social welfare*) and five issues were traditionally conservative (e.g., *a strong military*). As predicted, after reverse coding the 5 traditionally conservative issues the 10 items yielded a reliable composite score which we labeled “issue position” (α = .89; higher scores indicate more liberal positions).

From each participant’s zip code listed in the prescreening data, we were able to determine the percentage of people in that county that voted Democrat or Republican in the 2012 national election. Because values for percentage-voting-Democrat and percentage-voting-Republican were highly correlated, *r*(1269) = -.99, *p* < .001, we decided to use percentage-voting-Democrat (county blueness) values throughout.

Participants also indicated their voting intentions for the upcoming (2016) presidential election (*a Democratic candidate*, *a Republican candidate*, or *an independent candidate*). As in Study 1 we collapsed political identity into three categories (conservative, moderate, and liberal). Participants who did not specify either a Democratic or Republican candidate were excluded (*n* = 213), yielding a sample of 1,056 participants for analyses involving voting intentions.

## Results–Study 2

**Fig 2:**
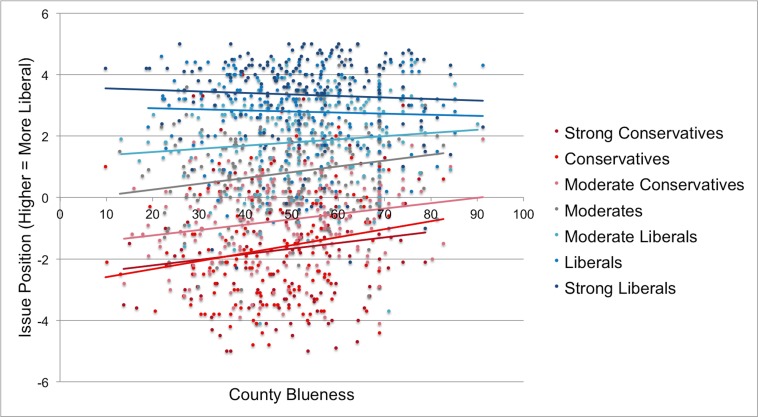
County blueness indicates percentage of participant’s county voting democrat in the 2012 presidential election. Lines are linear trendlines calculated separately for each level of political identity. If political ideology had the same meaning regardless of location the trendlines would be flat.

**Fig 3:**
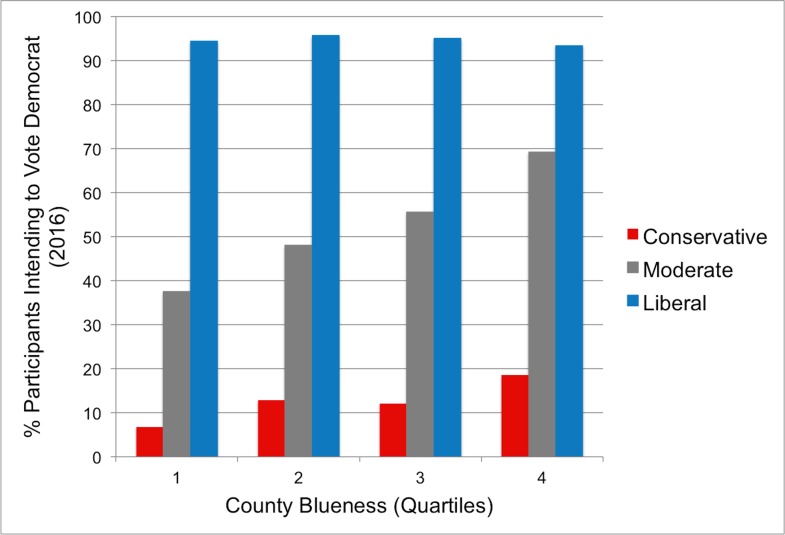
Voting intentions by county blueness for each political identity category. County blueness is divided into quartiles for visualization purposes, but was treated as a continuous variable in all analyses. If political ideology had the same meaning regardless of location all bars of the same color would be the same height.

In the second data set we observed support for the political reference point hypothesis at the county level. Running the same analysis as in Study 1 revealed a significant effect of county blueness, *R*^*2*^_*change*_ = .005, *F*(1, 1266) = 15.76, *p* < .001, such that bluer counties were associated with more liberal issue positions controlling for identity. Exploratory analyses revealed an interaction between political identity and county blueness, *R*^*2*^_*change*_ = .005, *F*(1, 1265) = 14.76, *p* < .001. Simple slope analyses indicated that county blueness was a significant predictor of policy positions for everyone except for liberals and strong liberals (Fig 2; Table F in S1 File). In addition, people in bluer (vs. redder) counties were more likely to say they intended to vote Democrat in the upcoming 2016 election, χ^2^(1) = 7.15, *p* = .007 (Fig 3).

## General discussion

These findings demonstrate that political identity, though typically understood as having universal meaning, is actually dependent on social context; from location to location ideological classifications mean different things. Although the effects we found were small (*R*^*2*^_*change*_ = .004 in Study 1, and *R*^*2*^_*change*_ = .005 in Study 2), we think they are meaningful for at least two reasons. First, *any* effect is notable given that political identity is often used as a proxy for issue position, and thus incremental predictive power should be difficult to come by. In particular, the fact that this effect is in the same direction across different samples is telling given that there were plausible reasons to expect no effect, or even the opposite effect.

**Fig 4:**
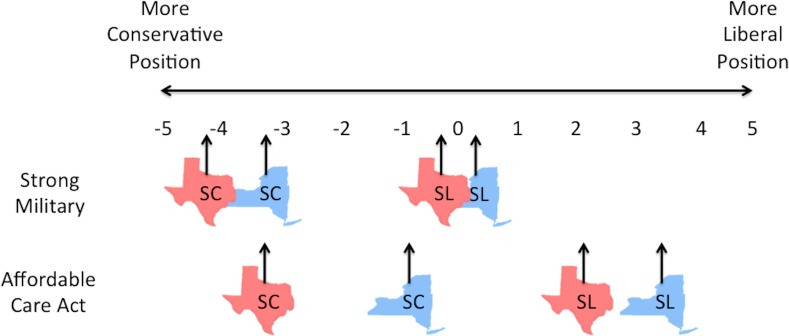
An illustrative example of the effect of location on policy position. SC = strong conservative identity; SL = strong liberal identity. Red Texas symbols represent people in the 100 reddest counties in America; Blue New York symbols represent people in the 100 bluest counties in America. One conservative and one liberal issue were chosen for illustrative purposes. Data are from Study 2.

Second, even small effect sizes can result in meaningful differences. For instance, the ANES data showed that *extremely conservative* people in Utah (24.9% blue) were reluctant to consider legalizing abortion even in cases of rape, whereas *extremely conservative* people in Hawaii (70.60% blue) were willing to consider legalizing abortion in a range of circumstances. In the second data set, when it came to their views on having a strong military, *moderates* in the bluest counties were effectively indistinguishable from *strong liberals* in the reddest counties (with *M*s of .33 and .38, respectively). In the 2016 Presidential election, a *moderate* in Philadelphia County, PA (85.2% blue) was more than 3 times as likely to indicate an intention to vote Democrat compared to a *moderate* in Brown County, TX (13.7% blue). Thus, those who identify in the exact same way on the political ideology spectrum may possess markedly different policy stances and preferences; meanwhile, people with different identities may hold identical or even ideologically reversed views (Fig 4).

It is important to consider that the effect of state blueness in the ANES datasets was more robust for some years than it was for others, possibly suggesting that people’s use of others around them as a political reference point might wax and wane depending on other time-sensitive influences. Presumably, the effect depends on the extent to which individuals use people “typical” of their location (in terms of sociopolitical characteristics) to calibrate their own political identity, so the effect may fluctuate to the extent that this behavior fluctuates. That said, the effect appears to be reliable given that it was present when considering the ANES data as a whole. Moreover, Study 2 replicated the effect using a targeted sample that included participants with all levels of political identification (including a large sample of those on the extremes) and with a distinct set of policy items.

All in all, we believe our findings highlight how the meaning of political identity is not objective, but rather socially determined–a point rarely considered by researchers or the lay public. Along these lines, our results may also align with theorizing about political ideology having both symbolic and operational components [24, 25], where the first refers to more abstract categories and stereotypes, and the latter refers to the more concrete policy positions. When individuals answer questions about political identification they might rely more on symbolic ideology, but when answering policy preference items they might rely more on operational ideology [5]. If the social environment impacts one of these ideologies more than the other, it may help account for why a growing number of studies, including the present one, find a disconnect between political identification and policy attitudes (e.g., [8, 26–29]).

On a related note, although the results we found highlight how one’s location affects the relationship between political identity and policy attitudes, we cannot make direct claims about causality. What exactly does one’s social environment affect–political identity, policy attitudes, both? The answer would seem to depend on which of these two is more susceptible to social influence. Existing literature on social identity–i.e., one’s sense of self based on social group membership [30, 31]–suggests that it can be malleable depending on the social context [32, 33], whereas many studies suggest that political stances, especially those grounded in moral convictions, are less flexible and may become even more strongly held when faced with contrasting viewpoints [34–36]. Thus, it may be that where individuals live more strongly affects their reported identity, but has less of an impact on their actual beliefs and stances. Future research that follows individuals who move from strongly blue to red, or red to blue, locations would help provide insight into exactly how people’s surroundings influence the relationship between political identity and policy stances.

Overall, our results suggest that researchers who rely on political identity as a proxy for classifying and estimating study participants’ beliefs and voting behavior may be using an oversimplified measure. Pollsters who administer specific questions to respondents based on their reported identity may be introducing error into their polling by not accounting for county-to-county variation in what those identities mean. Additionally, pollsters and the media often present polling results grouped into political identity clusters to provide a snapshot of attitudes across the political spectrum. But, again, this snapshot may be inaccurate unless the location of each pollee is taken into consideration. In sum, assessing political identity without considering location ignores the impact of social context.

Our findings also suggest that the animosity and disgust so commonly felt toward those on the other side of the political ideology spectrum may often be misplaced. Indeed, our results suggest that if a person feels hatred toward others simply based on how they identify on the political ideology spectrum, then in some circumstances, that hatred is actually aimed at someone with *the exact same policy stances*. Conversely, the ingroup favoritism commonly afforded those with the same political identity (e.g., [37]) may ironically involve giving preferential treatment to individuals who hold the opposing viewpoint on various issues. Overall, our findings suggest that it is important to consider that, oftentimes, it is not the policy preferences or the values that differ between people, but simply the labels they give themselves–labels that shift depending on their political reference point.

## Supporting information

S1 File **Fig A)** State blueness indicates percentage of participant’s state voting democrat in the 2012 presidential election. Lines are linear trendlines calculated separately for each level of political identity. Data are from Study 1. **Fig B)** State blueness indicates percentage of participant’s state voting democrat in the 2008 presidential election. Data are from Study 1. **Fig C)** State blueness indicates the percentage of each participant’s state voting Democrat in the 2008 presidential election. Lines are linear trendlines calculated separately for each level of political identity. Data are from the ANES. **Fig D)** State blueness indicates percentage of participant’s state voting democrat in the 2004 presidential election. Data are from the ANES. **Fig E)** State blueness indicates the percentage of each participant’s state voting Democrat in the 2004 presidential election. Lines are linear trendlines calculated separately for each level of political identity. Data are from the ANES. **Fig F)** State blueness indicates percentage of participant’s state voting democrat in the 2000 presidential election. Data are from the ANES. **Fig G)** State blueness indicates percentage of participant’s state voting democrat in the 2000 presidential election. Lines are linear trendlines calculated separately for each level of political identity. Data are from the ANES. **Fig H)** State blueness indicates percentage of participant’s state voting democrat in the 1996 presidential election. Data are from the ANES. **Fig I)** State blueness indicates percentage of participant’s state voting democrat in the 1996 presidential election. Lines are linear trendlines calculated separately for each level of political identity. Data are from the ANES. **Fig J)** State blueness indicates percentage of participant’s state voting democrat in the 1992 presidential election. Data are from the ANES. **Fig K)** State blueness indicates percentage of participant’s state voting democrat in the 1992 presidential election. Lines are linear trendlines calculated separately for each level of political identity. Data are from the ANES. **Fig L)** State blueness indicates percentage of participant’s state voting democrat in the 1988 presidential election. Data are from the ANES. **Fig M)** State blueness indicates percentage of participant’s state voting democrat in the 1988 presidential election. Lines are linear trendlines calculated separately for each level of political identity. Data are from the ANES. **Fig N)** State blueness indicates percentage of participant’s state voting democrat in the 1984 presidential election. Data are from the ANES. **Fig O)** State blueness indicates percentage of participant’s state voting democrat in the 1984 presidential election. Lines are linear trendlines calculated separately for each level of political identity. Data are from the ANES. **Fig P)** State blueness indicates percentage of participant’s state voting democrat in the 1980 presidential election. Data are from the ANES. **Fig Q)** State blueness indicates percentage of participant’s state voting democrat in the 1980 presidential election. Lines are linear trendlines calculated separately for each level of political identity. Data are from the ANES. **Fig R)** State blueness indicates percentage of participant’s state voting democrat in the 1976 presidential election. Data are from the ANES. **Fig S)** State blueness indicates percentage of participant’s state voting democrat in the 1976 presidential election. Lines are linear trendlines calculated separately for each level of political identity. Data are from the ANES. **Fig T)** State blueness indicates percentage of participant’s state voting democrat in the 1972 presidential election. Data are from the ANES. **Fig U)** State blueness indicates percentage of participant’s state voting democrat in the 1972 presidential election. Lines are linear trendlines calculated separately for each level of political identity. Data are from the ANES. **Fig V)** State blueness indicates percentage of participant’s state voting democrat in the 1968 presidential election. Data are from the ANES. **Fig W)** State blueness indicates the percentage of each participant’s state voting Democrat in the corresponding year’s presidential election. Data include all election years from 1972–2008. Lines are linear trendlines calculated separately for each level of political identity. Data are from the ANES. **Fig X)** State blueness indicates percentage of participant’s state voting democrat in the previous presidential election. Data include all election years from 1972–2008. Data are from the ANES. **Table A)** Descriptive statistics for issue position by political identity. Data are from Study 1. **Table B)** Unstandardized betas for simple slopes for issue position by state blueness at each level of political identity. Data are from Study 1. **Table C)** Key results from stepwise regressions conducted for each issue individually. The *R*^*2*^_*change*_ statistic is for Step 2 of a stepwise regression in which issue stance is predicted from political orientation (Step 1), location (Step 2) and their interaction (Step 3). Issues are recoded such that scores are on a scale of 0 to 1, and higher scores indicate more liberal positions. Data are from Study 1. **Table D)** Number of participants by state color and political identity. For political identity, 1 **=** strong conservative, 2 **=** conservative, 3 **=** moderate conservative, 4 **=** moderate, 5 **=** moderate liberal, 6 **=** liberal, 7 **=** strong liberal. Data are from Study 2. **Table E)** Descriptive statistics for issue position by political identity. Data are from Study 2. **Table F)** Unstandardized betas for simple slopes for issue position by state blueness at each level of political identity. Data are from Study 2. **Table G)** Key results from stepwise regressions conducted for each issue individually. The *R*^*2*^_*change*_ statistic is for Step 2 of a stepwise regression in which issue stance is predicted from political orientation (Step 1), location (Step 2) and their interaction (Step 3). Issues are recoded such that higher scores indicate more liberal positions. Data are from Study 2. **Table H)** Items included in “issue position” variable, by year. Data are from the ANES. **Table I)** Issue position data for the year 2008. Unstandardized betas for simple slopes for issue position by state blueness at each level of political identity. Statistically significant results are highlighted in bold. Data are from the ANES. **Table J)** Voting data for the year 2008. Unstandardized betas for simple effects for voting by state blueness at each level of political identity. Statistically significant results are highlighted in bold. Data are from the ANES. **Table K)** Issue position data for the year 2004. Unstandardized betas for simple slopes for issue position by state blueness at each level of political identity. Statistically significant results are highlighted in bold. Data are from the ANES. **Table L)** Voting data for the year 2004. Unstandardized betas for simple effects for voting by state blueness at each level of political identity. Statistically significant results are highlighted in bold. Data are from the ANES. **Table M)** Issue position data for the year 2000. Unstandardized betas for simple slopes for issue position by state blueness at each level of political identity. Statistically significant results are highlighted in bold. Data are from the ANES. **Table N)** Voting data for the year 2000. Unstandardized betas for simple effects for voting by state blueness at each level of political identity. Statistically significant results are highlighted in bold. Data are from the ANES. **Table O)** Issue position data for the year 1996. Unstandardized betas for simple slopes for issue position by state blueness at each level of political identity. Statistically significant results are highlighted in bold. Data are from the ANES. **Table P)** Voting data for the year 1996. Unstandardized betas for simple effects for voting by state blueness at each level of political identity. Statistically significant results are highlighted in bold. Data are from the ANES. **Table Q)** Issue position data for the year 1992. Unstandardized betas for simple slopes for issue position by state blueness at each level of political identity. Statistically significant results are highlighted in bold. Data are from the ANES. **Table R)** Voting data for the year 1992. Unstandardized betas for simple effects for voting by state blueness at each level of political identity. Statistically significant results are highlighted in bold. Data are from the ANES. **Table S)** Issue position data for the year 1988. Unstandardized betas for simple slopes for issue position by state blueness at each level of political identity. Statistically significant results are highlighted in bold. Data are from the ANES. **Table T)** Voting data for the year 1988. Unstandardized betas for simple effects for voting by state blueness at each level of political identity. Statistically significant results are highlighted in bold. Data are from the ANES. **Table U)** Issue position data for the year 1984. Unstandardized betas for simple slopes for issue position by state blueness at each level of political identity. Statistically significant results are highlighted in bold. Data are from the ANES. **Table V)** Voting data for the year 1984. Unstandardized betas for simple effects for voting by state blueness at each level of political identity. Statistically significant results are highlighted in bold. Data are from the ANES. **Table W)** Issue position data for the year 1980. Unstandardized betas for simple slopes for issue position by state blueness at each level of political identity. Statistically significant results are highlighted in bold. Data are from the ANES. **Table X)** Voting data for the year 1980. Unstandardized betas for simple effects for voting by state blueness at each level of political identity. Statistically significant results are highlighted in bold. Data are from the ANES. **Table Y)** Issue position data for the year 1976. Unstandardized betas for simple slopes for issue position by state blueness at each level of political identity. Statistically significant results are highlighted in bold. Data are from the ANES. **Table Z)** Voting data for the year 1976. Unstandardized betas for simple effects for voting by state blueness at each level of political identity. Statistically significant results are highlighted in bold. Data are from the ANES. **Table AA)** Issue position data for the year 1972. Unstandardized betas for simple slopes for issue position by state blueness at each level of political identity. Statistically significant results are highlighted in bold. Data are from the ANES. **Table BB)** Voting data for the year 1972. Unstandardized betas for simple effects for voting by state blueness at each level of political identity. Statistically significant results are highlighted in bold. Data are from the ANES. **Table CC)** Issue position data for all election years combined (1972 through 2012). Unstandardized betas for simple slopes for issue position by state blueness at each level of political identity. Statistically significant results are highlighted in bold. Data are from the ANES. **Table DD)** Voting data for all election years combined (1972 through 2012). Unstandardized betas for simple effects for voting by state blueness at each level of political identity. Statistically significant results are highlighted in bold. Data are from the ANES. **Table EE)** Summary of regression results for issue position. Data are from the ANES. **Table FF)** Summary of regression results for voting behaviors. Data are from the ANES.
(DOCX)
