# Supplementary material for: The political reference point: How geography shapes political identity
Source: PLoS One. 2017 Feb 16;12(2):e0171497. doi: 10.1371/journal.pone.0171497 (PMC5312959; doi:10.1371/journal.pone.0171497)
Supplement: S1 File — Fig A) State blueness indicates percentage of participant’s state voting democrat in the 2012 presidential election. Lines are linear trendlines calculated separately for each level of political identity. Data are from Study 1. Fig B) State blueness indicates percentage of participant’s state voting democrat in the 2008 presidential election. Data are from Study 1. Fig C) State blueness indicates the percentage of each participant’s state voting Democrat in the 2008 presidential election. Lines are linear trendlines calculated separately for each level of political identity. Data are from the ANES. Fig D) State blueness indicates percentage of participant’s state voting democrat in the 2004 presidential election. Data are from the ANES. Fig E) State blueness indicates the percentage of each participant’s state voting Democrat in the 2004 presidential election. Lines are linear trendlines calculated separately for each level of political identity. Data are from the ANES. Fig F) State blueness indicates percentage of participant’s state voting democrat in the 2000 presidential election. Data are from the ANES. Fig G) State blueness indicates percentage of participant’s state voting democrat in the 2000 presidential election. Lines are linear trendlines calculated separately for each level of political identity. Data are from the ANES. Fig H) State blueness indicates percentage of participant’s state voting democrat in the 1996 presidential election. Data are from the ANES. Fig I) State blueness indicates percentage of participant’s state voting democrat in the 1996 presidential election. Lines are linear trendlines calculated separately for each level of political identity. Data are from the ANES. Fig J) State blueness indicates percentage of participant’s state voting democrat in the 1992 presidential election. Data are from the ANES. Fig K) State blueness indicates percentage of participant’s state voting democrat in the 1992 presidential election. Lines are linear [file pone.0171497.s001.docx]

**Supplemental Information**

**Study 1**

**Method**

In Study 1 we examined whether specific political identities (e.g., “strong conservative”) are associated with different positions on issues depending on one’s state of residence. To do so we used data from the publicly available American National Election Survey (ANES) dataset. For political identity we used the “IDEOLOGY: Liberal-Conservative Scale” item (VCF0803; 1 = Extremely liberal, 2 = Liberal, 3 = Slightly liberal, 4 = Moderate, middle of the road, 5 = Slightly conservative, 6 = Conservative, 7 = Extremely conservative). Responses were reverse coded so that higher scores indicated greater liberalism. For views on political issues we selected all issues that were answered by participants in 2012, and that had a non-categorical rating scale. From these we removed redundant items, maintaining the item with more response options (e.g., “strength of position” items were included over “favor vs. oppose” items) resulting in a set of 9 items (VCF0806, VCF0809, VCF0830, VCF0838, VCF0839, VCF0843, VCF0867a, VCF0876a, VCF0877a). We then reverse-coded items to ensure that higher values indicated more liberal positions, and converted responses to a 0-1 scale (by dividing by the number of response options) to account for variation in the number of response options. From these values we computed a composite score which we labeled “issue position” (α = .79). Participants were included in analyses if they responded to all 9 issues (excluding “N/A” or “I don’t know”) and the identity item. This yielded a sample of 1809 participants for analyses involving issues.

From each participant’s state of residence we were able to determine the percentage of people in that state that voted Democrat or Republican in the 2012 national election (n.b., we used state instead of congressional district because congressional district boundaries, numbers, and labels fluctuate from year to year). Values for percentage-voting-Democrat and percentage-voting-Republican were highly correlated, *r*(1809) = -.99, *p* < .001, so we again used percentage-voting-Democrat (state blueness) values throughout.

The ANES dataset provided us with the opportunity to assess whether political identities were associated with different voting behavior depending on one’s state of residence. To assess voting behavior we used the “ELECTION: Vote for President – Major Parties” item (VCF0704a; 1 = *Democrat*, 2 = *Republican*), and recoded responses such that higher values corresponding to voting Democrat. For analyses involving voting behavior we used state blueness values from the previous election (i.e., 2008) to avoid redundancy between the state blueness predictor variable and the voting outcome variable. For analyses of voting intentions we collapsed political identity into three categories (conservative, moderate, and liberal) as we were primarily interested in comparing identity-consistent and identity-inconsistent voting across states. Participants were included in analyses if they voted for a Democratic or Republican candidate and responded to the identity item. This yielded a sample of 3862 participants for analyses involving voting.

**Results**

Descriptive statistics are listed in Table A. To test whether political identity was associated with different issue positions depending on state blueness we ran a stepwise regression predicting issue position from political identity (centered; Step 1), state blueness (centered; Step 2) and their interaction (Step 3). This analysis revealed a significant effect of political identity, *R^2^* = .442, *SE* = .115, *F*(1, 1807) = 1430.73, p < .001, such that more liberal identities were associated with more liberal issue positions. It also revealed significant effect of state blueness, *R^2^_change_* = .004, *F*(1, 1806) = 12.32, *p* < .001, such that bluer counties were associated with more liberal issue positions controlling for identity. We observed a significant interaction between political identity and state blueness, *R^2^_change_*= .002, *F*(1, 1805) = 8.11, *p* = .004. Computing the simple slopes at different levels of political identity reveals that for conservatives and moderates, but not liberals, higher levels of state blueness are associated with more liberal issue positions (Fig. A, Table B). Results for individual issues are listed in Table C. Excluding people from counties with >90% voting democrat or <10% voting democrat yields similar results, *R^2^_change_* = .003, *F*(1, 1801) = 8.73, *p* = .003.

Next, we ran a stepwise logistic regression predicting voting behavior from political identity (dummy coded; Step 1), state blueness (centered; Step 2) and their interaction (Step 3). The main effect of political identity was significant, χ2(2) = 1670.66, p < .001, as was the main effect of state blueness, χ2(1) = 9.84, p =.002. This time we did not observe a significant interaction, χ2(2) = 2.31, p =.315. Examining the simple effects, we observed that for moderates, the effect of state blueness on voting was significant B = .026, SE = .008, p = .002. Increasing state blueness by 10% was associated with being 1.26 times more likely to vote democrat. For conservatives, the effect of state blueness on voting was not significant, B = .013, SE = .008, p = .112. Increasing state blueness by 10% was associated with being 1.13 times more likely to vote Democrat. For liberals, the effect of state blueness on voting was not significant, B = .001, SE = .016, p =.957. Increasing state blueness by 10% was associated with being 1.01 times as likely to vote Democrat (Fig B).

Study 2

**Method**

In Study 2 we assessed the same question as in Study 1 with a sample collected specifically to test this hypothesis. Here, we were able to assess “blueness” at the county level, allowing a more nuanced test of our main question. We also did targeted recruiting of participants in order to ensure that the extremes of political identity (i.e., *strong conservatives* and *strong liberals*) were well-represented. Finally, this study also allowed us to test the generalizability of our previous results by testing different political issues.

For each position on a political identity pre-screening measure (1 = *strong conservative*, 2 = *conservative*, 3 = *moderate conservative*, 4 = *moderate*, 5 = *moderate liberal*, 6 = *liberal*, 7 = *strong liberal*) we recruited 100 mTurk workers from red states and 100 from blue states. At the beginning of the survey we included an instructional manipulation check (*20*), to determine whether participants were paying attention to written instructions. Participants who failed the attention check twice were prevented from completing the study. Of 1,400 participants who were recruited, a total of 1,349 participants began the survey and 1,269 finished all relevant items. This sample of 1,269 people was used in analyses involving issue position (*M*_age_ = 39.80, *SD*_age_ = 12.94, 672 female; see Table D for number of participants in each category).

From each participant’s zip code listed in the prescreening data, we were able to determine the percentage of people in that county that voted Democrat or Republican in the 2012 national election. Because values for percentage-voting-Democrat and percentage-voting-Republican were highly correlated, *r*(1269) = -.99, *p* < .001, we decided to use percentage-voting-Democrat (county blueness) values throughout.

Participants indicated their position (-*5 = strongly oppose, 5 = strongly in favor*) on 10 political issues. Five issues were traditionally liberal (*women’s reproductive rights*, *same sex marriage*, *social welfare*, *affordable care act*, *raising the minimum wage*) and five issues were traditionally conservative (*a strong military*, *capital punishment*, *school prayer*, *off-shore drilling*, *enhanced interrogation techniques*). As predicted, after reverse coding the 5 traditionally conservative issues the 10 items yielded a reliable composite score which we labeled “issue position” (α = .89; higher scores indicate more liberal positions).

Participants also indicated their voting intentions for the upcoming (2016) presidential election (*a Democratic candidate, a Republican candidate,* or *an independent candidate*). As in Study 1 we collapsed political identity into three categories (conservative, moderate, and liberal). Participants who did not specify either a Democratic or Republican candidate were excluded (*n* = 213), yielding a sample of 1,056 participants for analyses involving voting intentions.

In addition, participants completed a number of measures that were not directly relevant to the current research question. For each of the 10 political issues participants indicated their estimates of the position taken by the “average person,” the “average liberal,” and the “average conservative.” Participants also read a brief article about Obamacare and answered 10 questions assessing the hostile media effect (*21*). Participants also completed demographic measures of religious affiliation, ethnicity, political affiliation, and political identity.

**Results**

Descriptive statistics are listed in Table E. To test whether political identity was associated with different issue positions depending on county blueness we ran a stepwise regression predicting issue position from political identity (centered; Step 1), county blueness (centered; Step 2) and their interaction (Step 3). This analysis revealed a significant effect of political identity, *R^2^* = .587, *SE* = 1.53, *F*(1, 1267) = 1799.95, p < .001, such that more liberal identities were associated with more liberal issue positions. It also revealed significant effect of county blueness, *R^2^_change_* = .005, *F*(1, 1266) = 15.76, *p* < .001, such that bluer counties were associated with more liberal issue positions controlling for identity. These main effects were qualified by a significant interaction between political identity and county blueness, *R^2^_change_*= .005, *F*(1, 1265) = 14.76, *p* < .001. This interaction indicates that the relationship between issue position and county blueness changes depending on political identity. Computing the simple slopes at different levels of political identity reveals that for everyone except for liberals and strong liberals, higher levels of county blueness are associated with more liberal issue positions (Fig. 2 in Main Document, Table F). Results for individual issues are listed in Table G. Excluding people from states with >90% voting democrat or <10% voting democrat yields similar results, *R^2^_change_* = .005, *F*(1, 1260) = 16.05, *p* < .001.

Next, we ran a stepwise logistic regression predicting voting intentions from political identity (dummy coded; Step 1), county blueness (centered; Step 2) and their interaction (Step 3). The main effect of political identity was significant, χ^2^(2) = 743.46, *p* < .001, as was the main effect of county blueness, χ^2^(1) = 7.15, *p* =.007. We also observed a marginally significant interaction, χ^2^(2) = 5.16, *p* =.076. Examining the simple effects, we observed that for conservatives, the effect of county blueness on voting was significant *B* = .022, *SE* = .011, *p* = .04. Increasing county blueness by 10% was associated with being 1.22 times more likely to vote democrat. For moderates, the effect of county blueness on voting was significant, *B* = .036, *SE* = .013, *p* = .007. Increasing county blueness by 10% was associated with being 1.36 times more likely to vote Democrat. For liberals, the effect of county blueness on voting was not statistically significant, *B* = -.001, *SE* = .013, *p* =.682. Increasing county blueness by 10% was associated with being .95 times as likely to vote Democrat (Fig. 3 in Main Document).

ANES DATA (1972 -2008)

**Method**

We also tested these effects using ANES data from previous Presidential election years going back to 1972 (years prior to 1972 do not include the political identity variable).

Political identity was measured in the same manner as in Study 1. To compute the issue position score for each year we first identified “ISSUES” items in the ANES dataset that were answered by participants in at least one year between 1972 and 2008, and that had a non-categorical rating scale. From these we removed redundant items and items that were weakly related to political identity (i.e., it was ambiguous which positions were more liberal). These decisions were made prior to data analysis, and yielded a set of 12 items (VCF0806, VCF0809, VCF0814, VCF0830, VCF0834, VCF0837, VCF0838, VCF0839, VCF0843, VCF0867a, VCF0876a, VCF0877a). We then reverse-coded items to ensure that higher values indicated more liberal positions, and converted responses to a 0-1 scale (by dividing by the number of response options) to account for variation in the number of response options. From these values we computed a composite score (“issue position”) for each year, including all items available for that year (αs between .57 and .79). Participants were included in analyses if they responded to all of the issues included in their year (excluding “N/A” or “I don’t know”) and the identity item. A summary of the items included in the issue position variable for each year is provided in Table H.

As in Study 1 were able to determine the percentage of people in each participant’s state that voted Democrat or Republican in the Presidential election. Values for percentage-voting-Democrat and percentage-voting-Republican were highly correlated, *r*(8336) = -.99, *p* < .001, so we again used percentage-voting-Democrat (state blueness) values throughout. For analyses with issue position as the dependent variable we used state blueness values from the corresponding year (e.g., issue position scores from 2008 were predicted from state blueness scores from 2008).

Voting behavior was measured in the same manner as in Study 1. For analyses involving voting behavior we used state blueness values from the previous election year (e.g., issue position scores from 2008 were predicted from state blueness scores from 2004). This decision was made to avoid redundancy between the state blueness predictor variable and the voting outcome variable.

**Results**

First, we tested whether political identity was associated with different issue positions depending on state blueness. For each year we ran a stepwise regression predicting issue position from political identity (centered within year; Step 1), state blueness (centered within year; Step 2) and their interaction (Step 3). We also conducted this analysis using data from all years combined, this time centering political identity and state blueness within the entire sample.

We also tested whether political identity was associated with different voting behavior depending on state blueness. For each year we ran a stepwise logistic regression predicting voting behavior from political identity (dummy coded; Step 1), state blueness (centered within year; Step 2) and their interaction (Step 3). We also conducted this analysis using data from all years combined, this time centering state blueness within the entire sample (Figs. C – X, Tables I – FF).


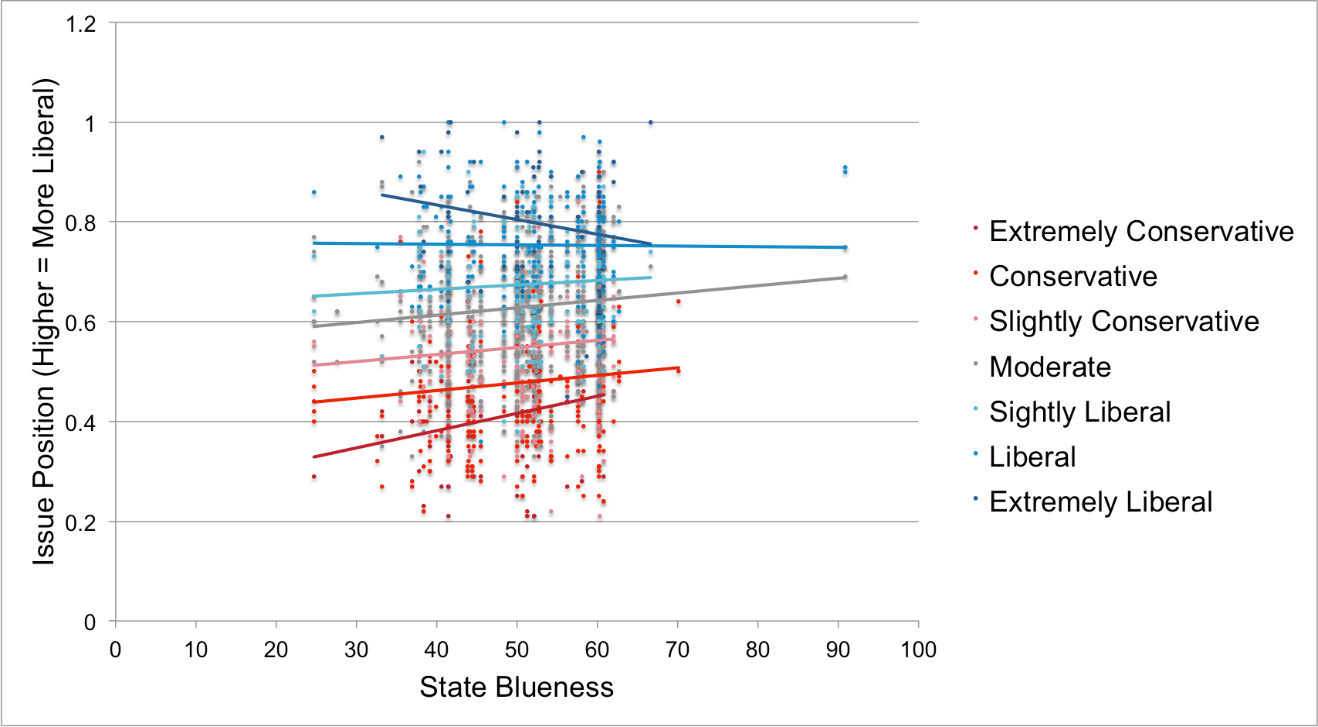


Figure A


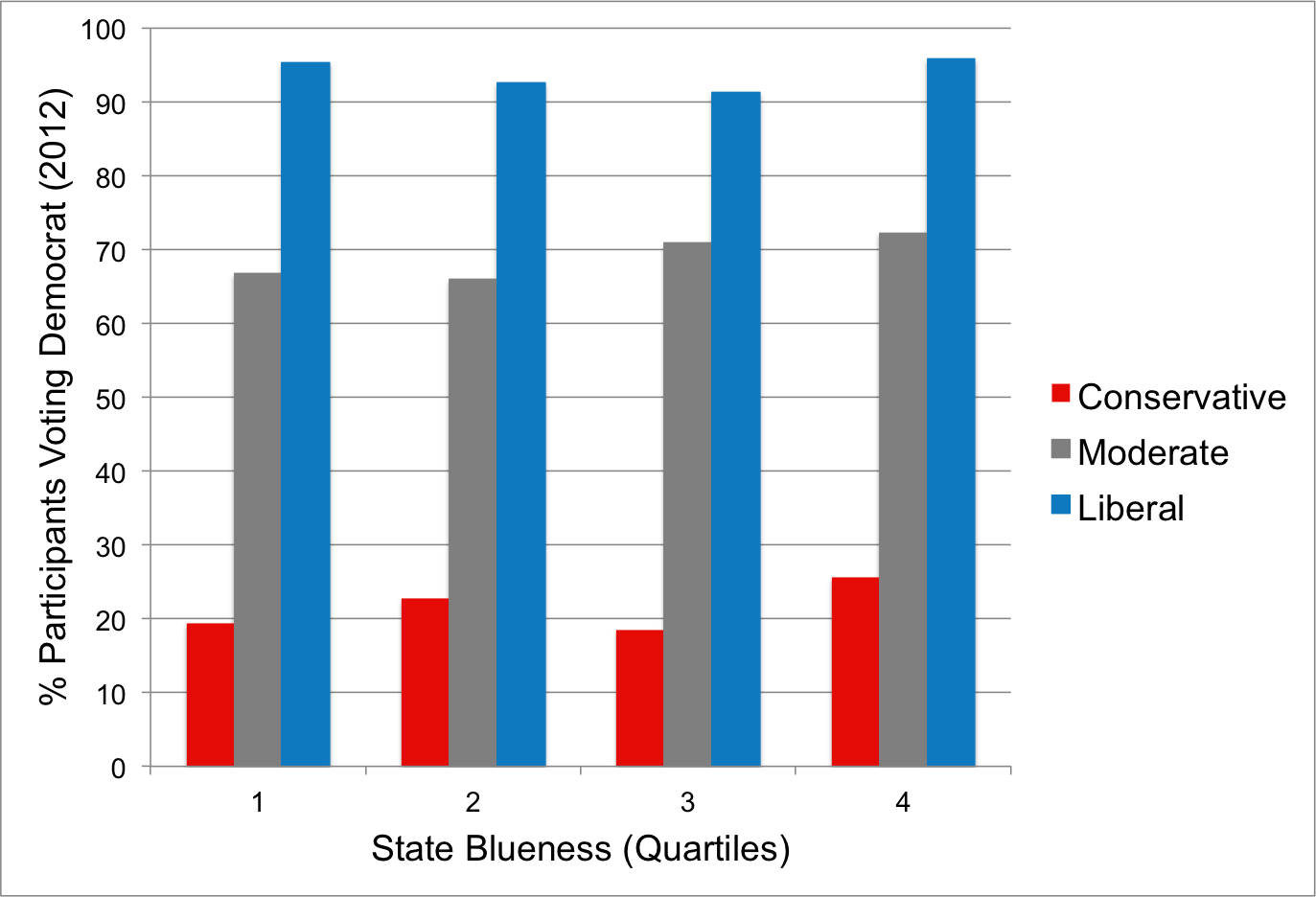


Figure B

**
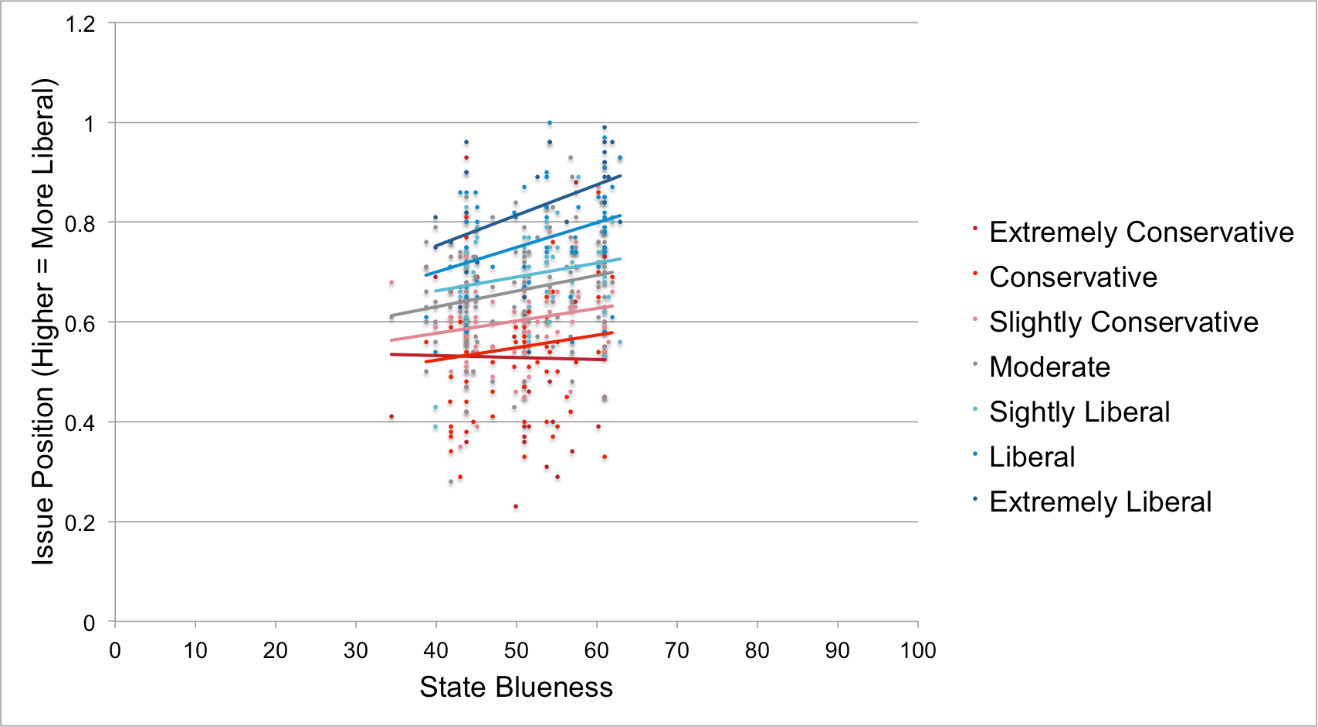
**

Figure C


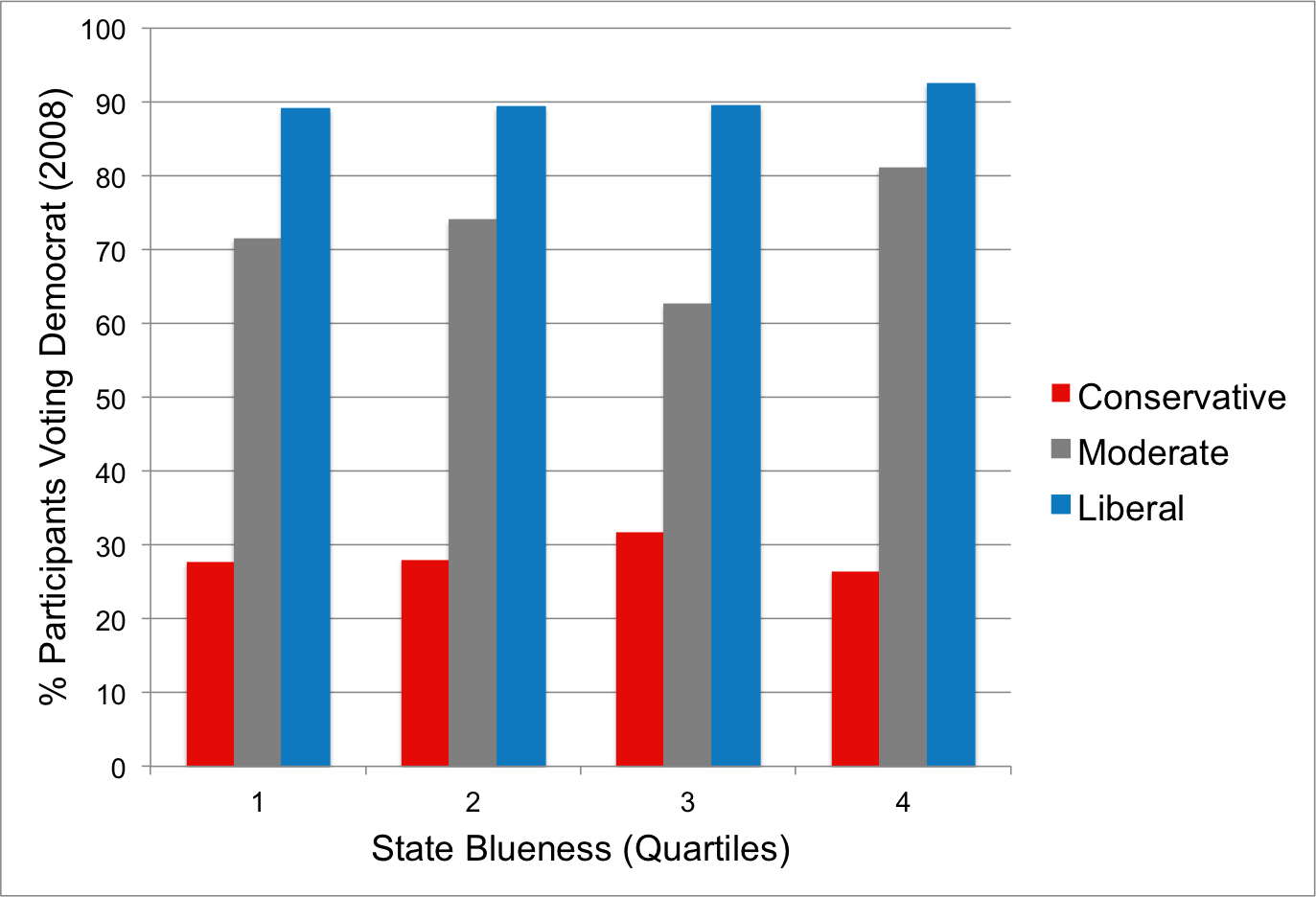


Figure D

**
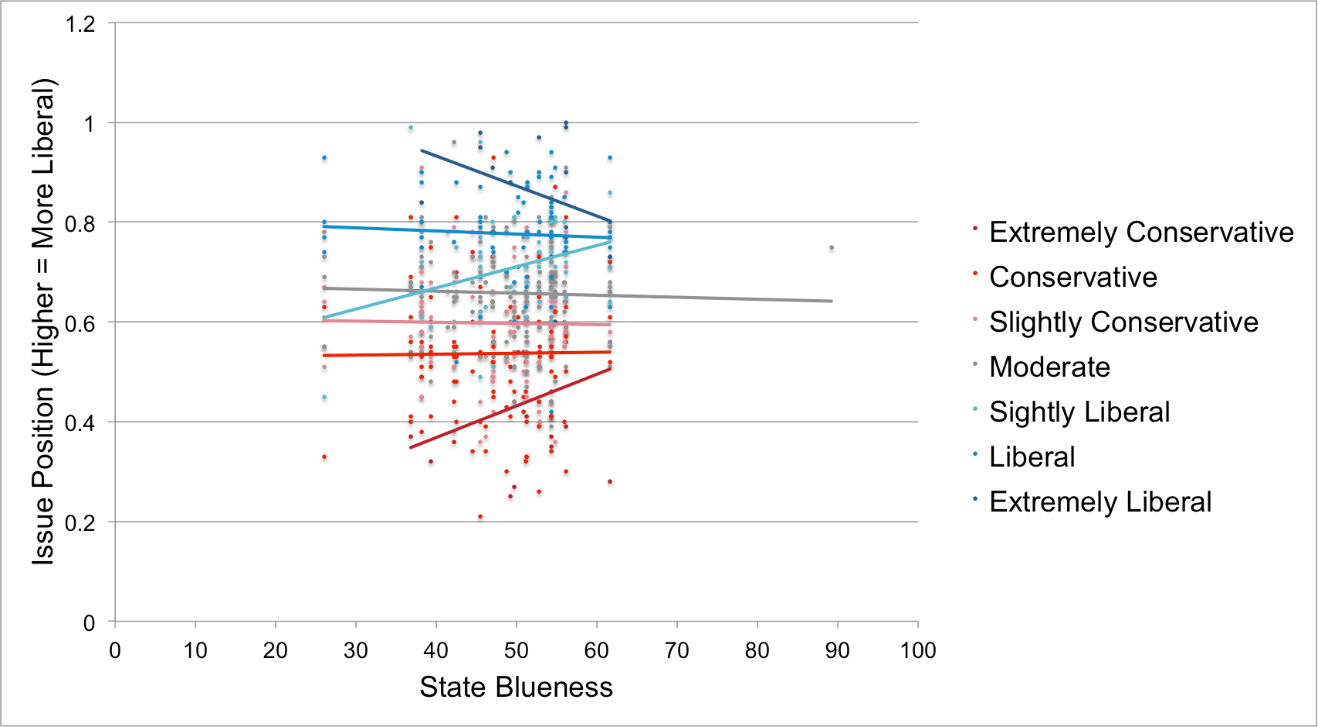
**

Figure E


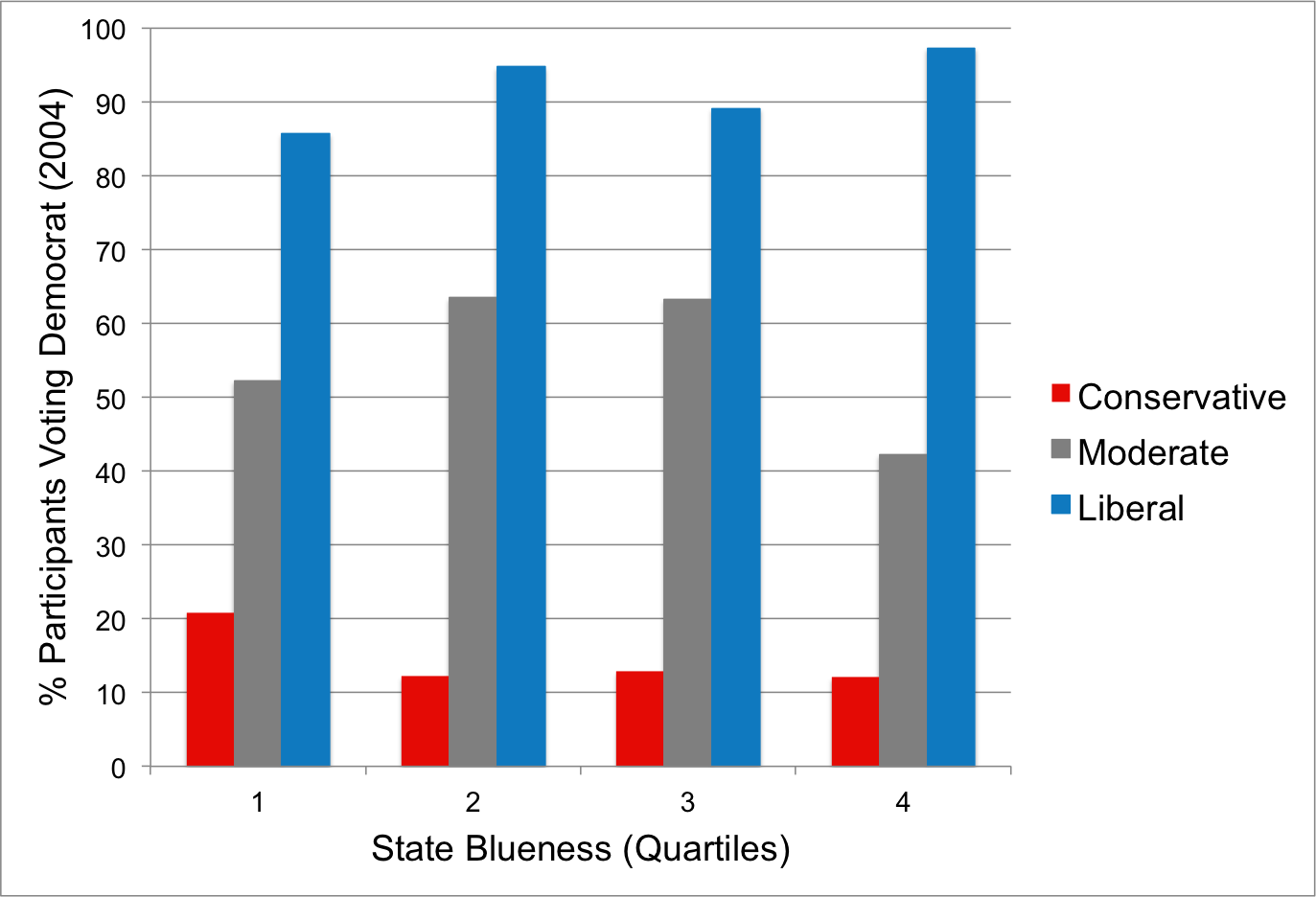


Figure F

**
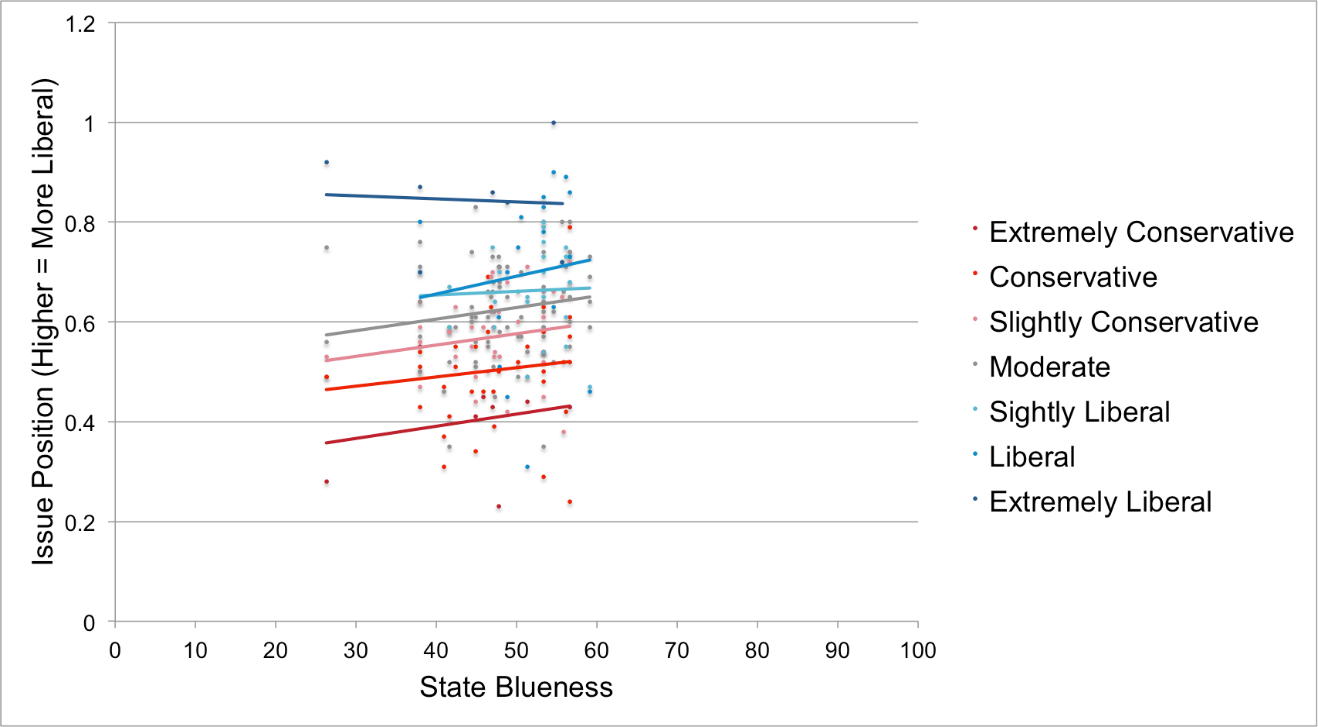
**

Figure G


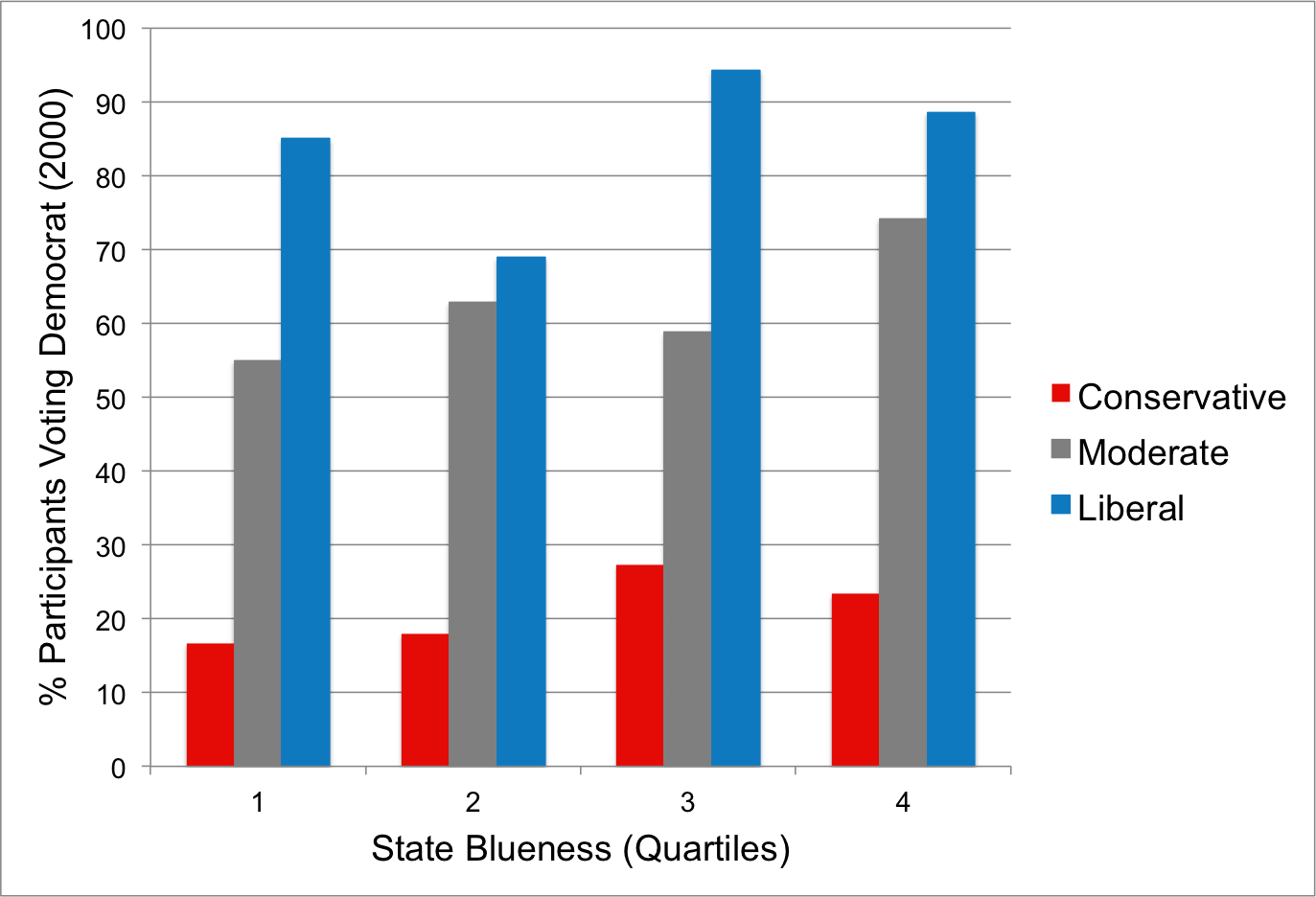


Figure H

**
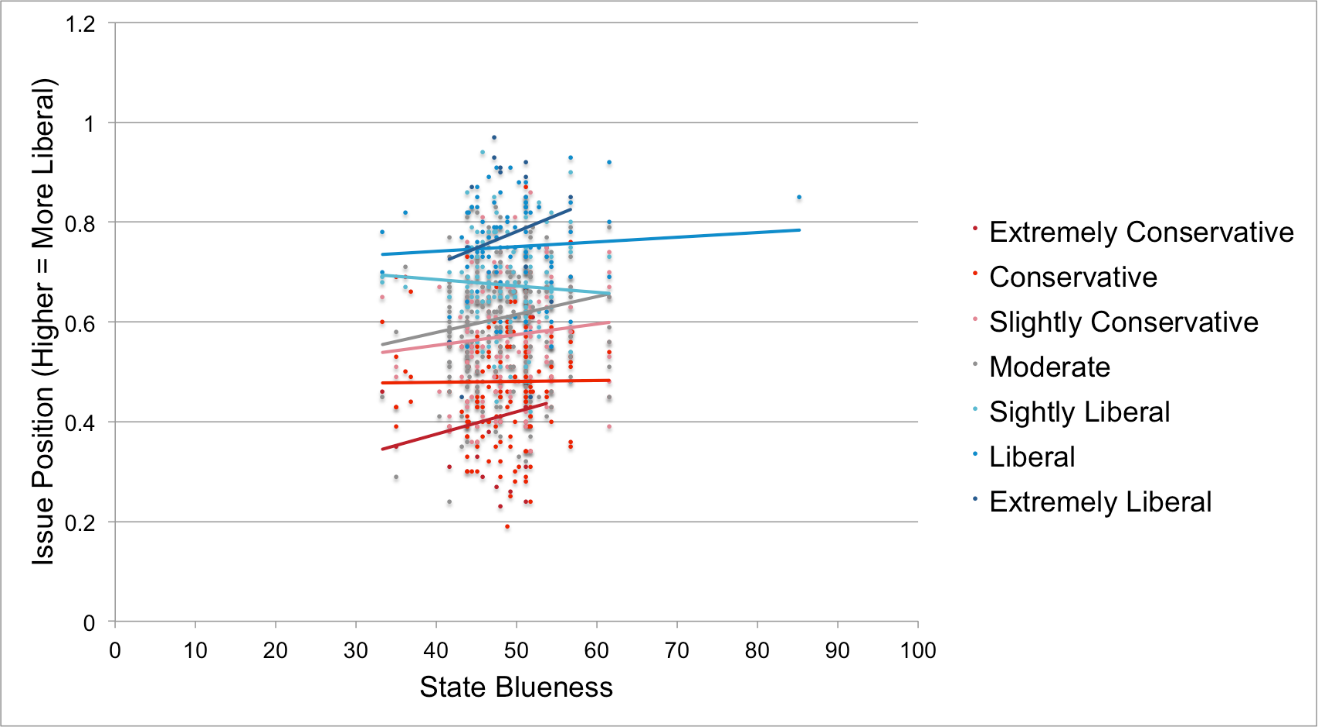
**

Figure I

**
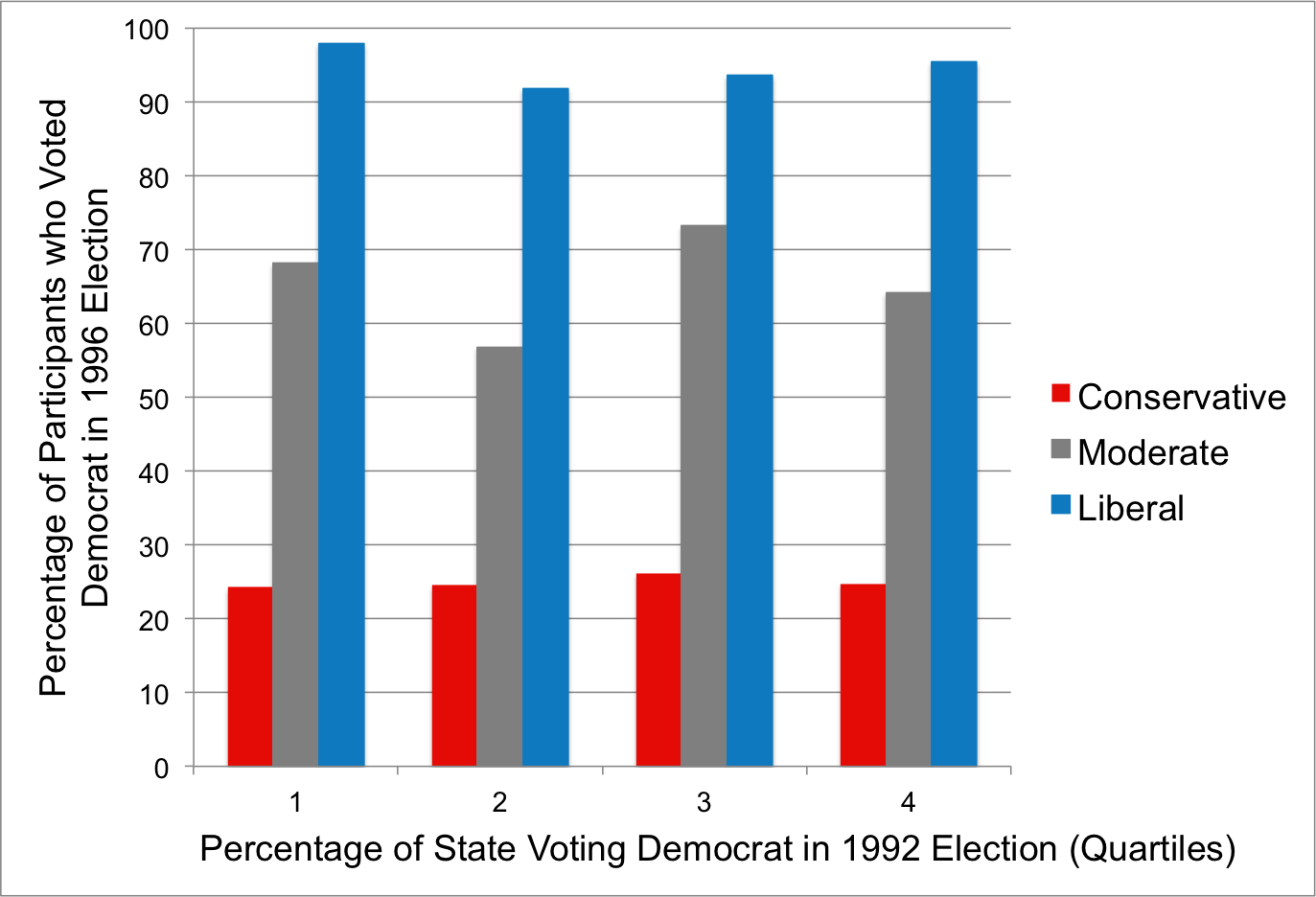
**

Figure J

**
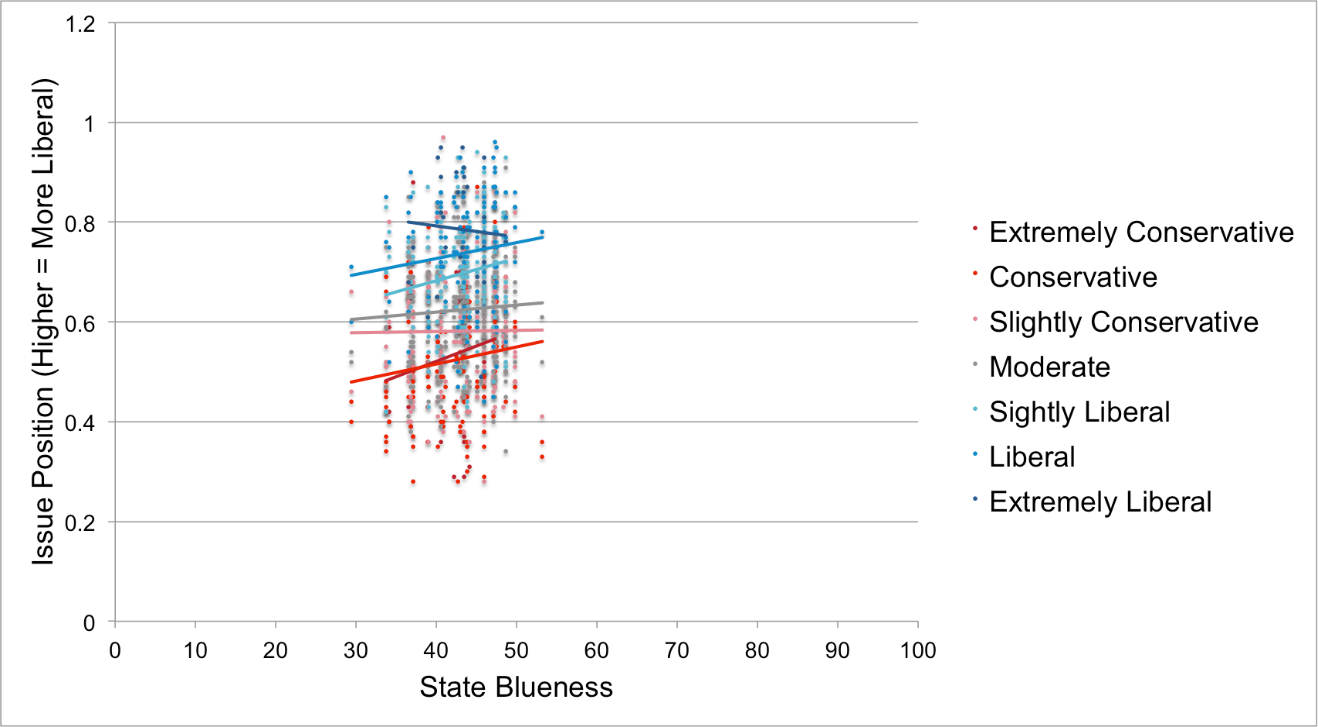
**

**Figure K**


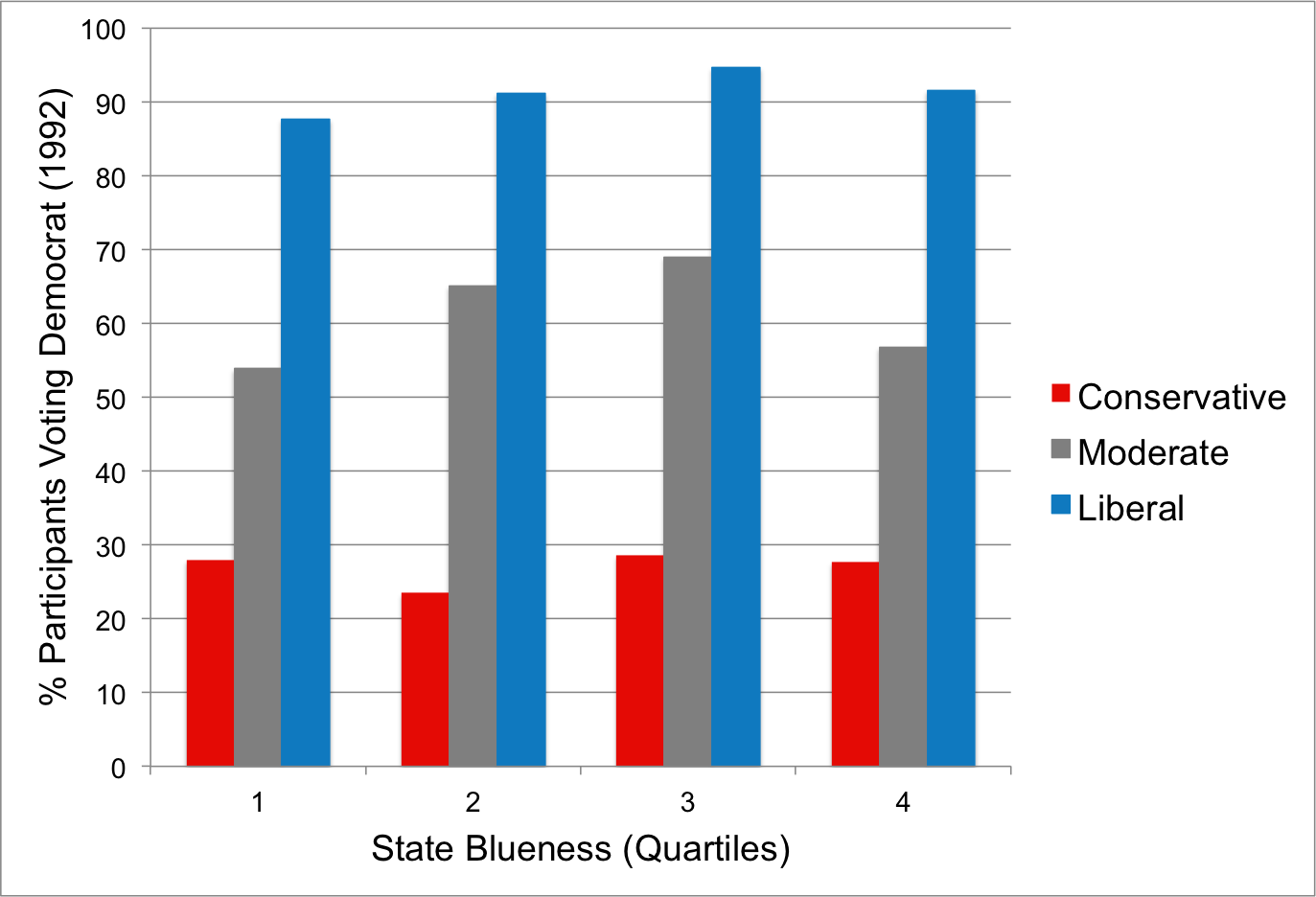


Figure L

**
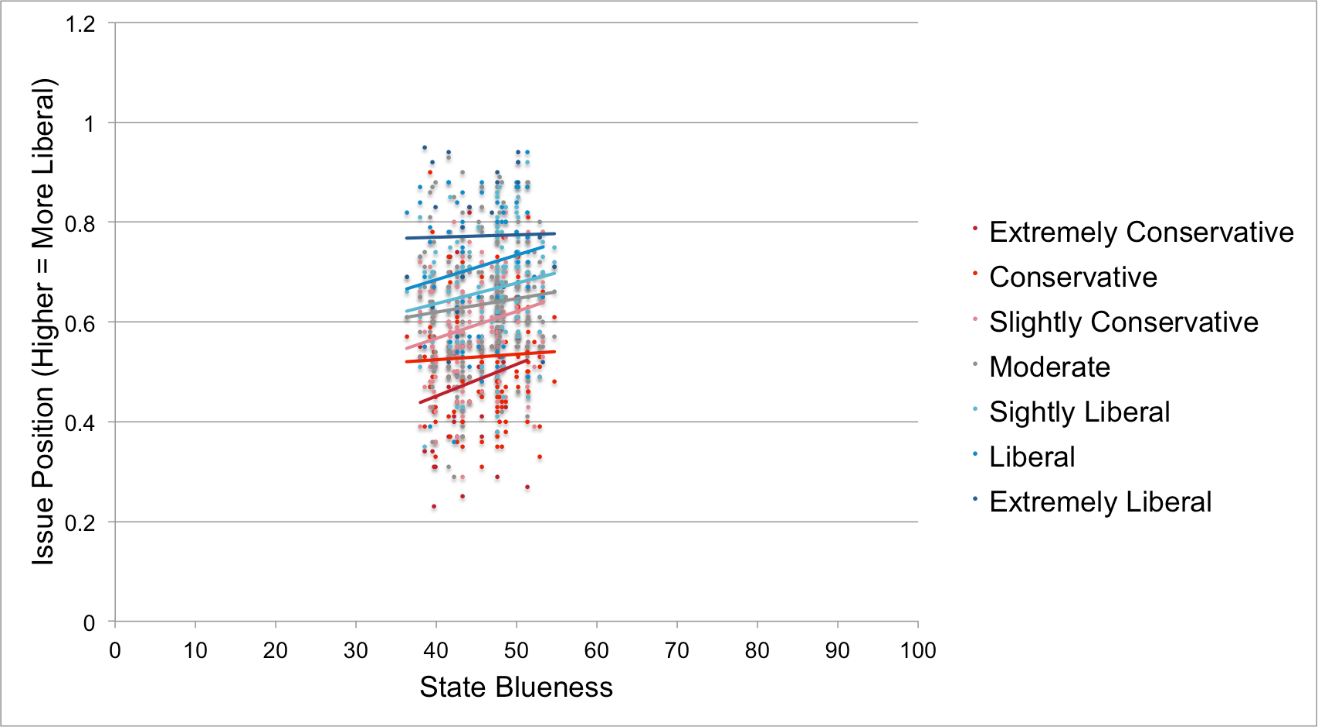
**

Figure M


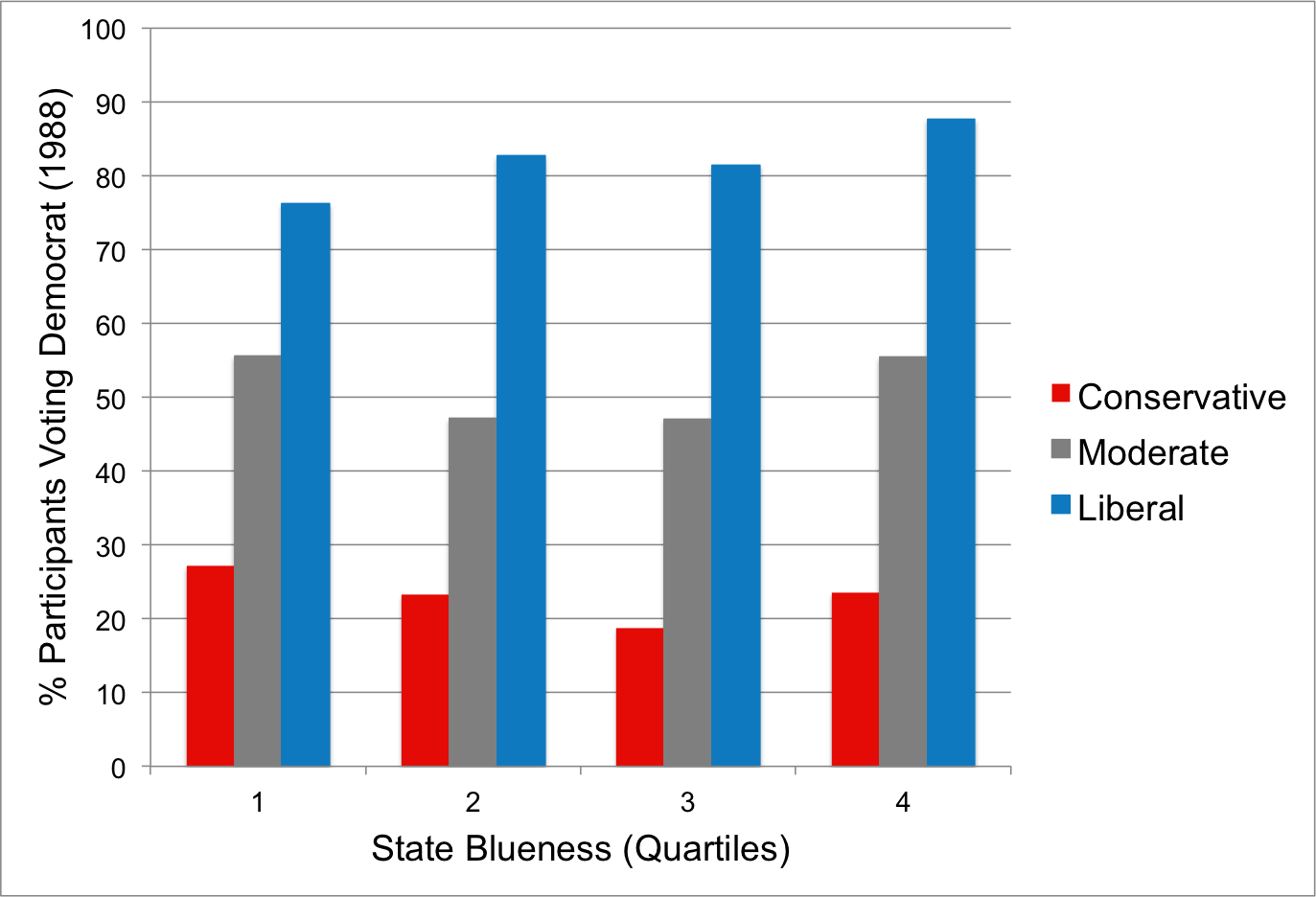


Figure N

**
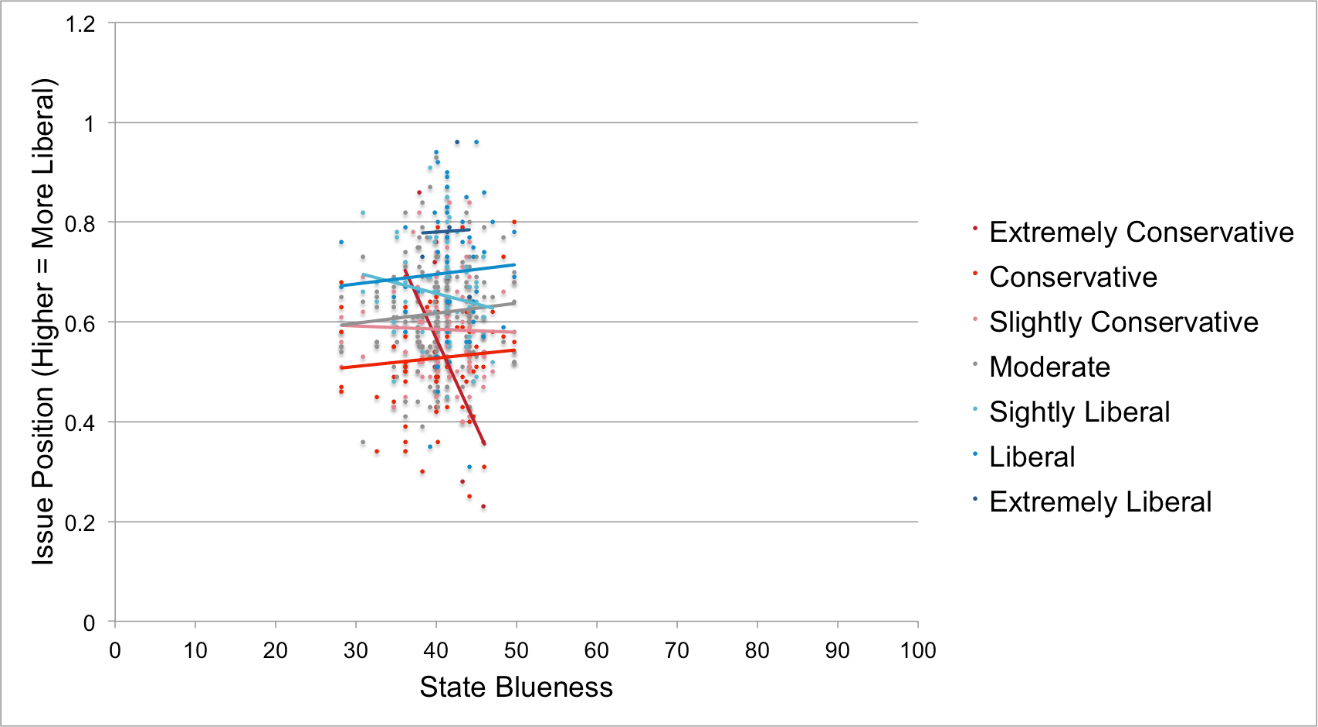
**

Figure O


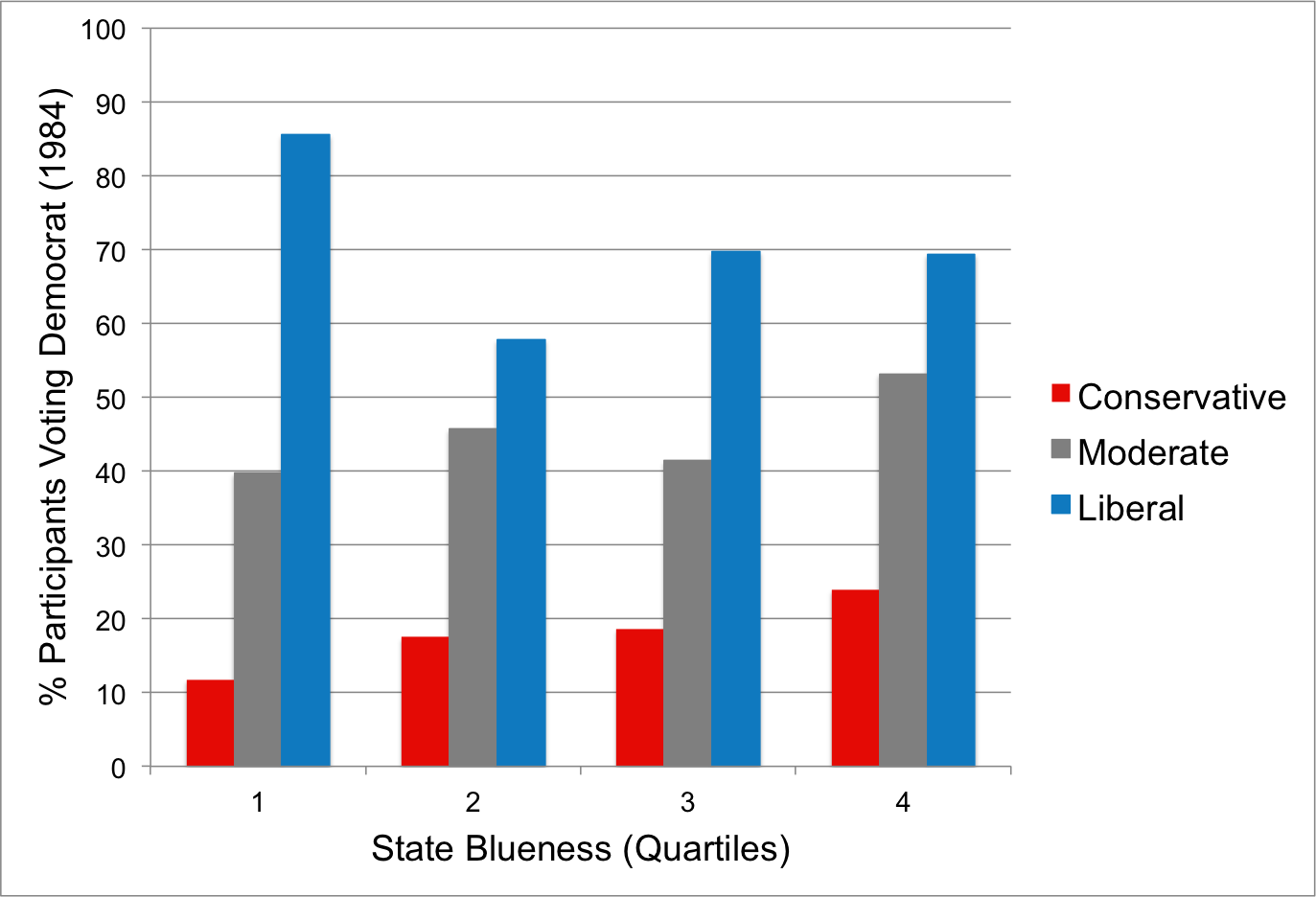


Figure P

**
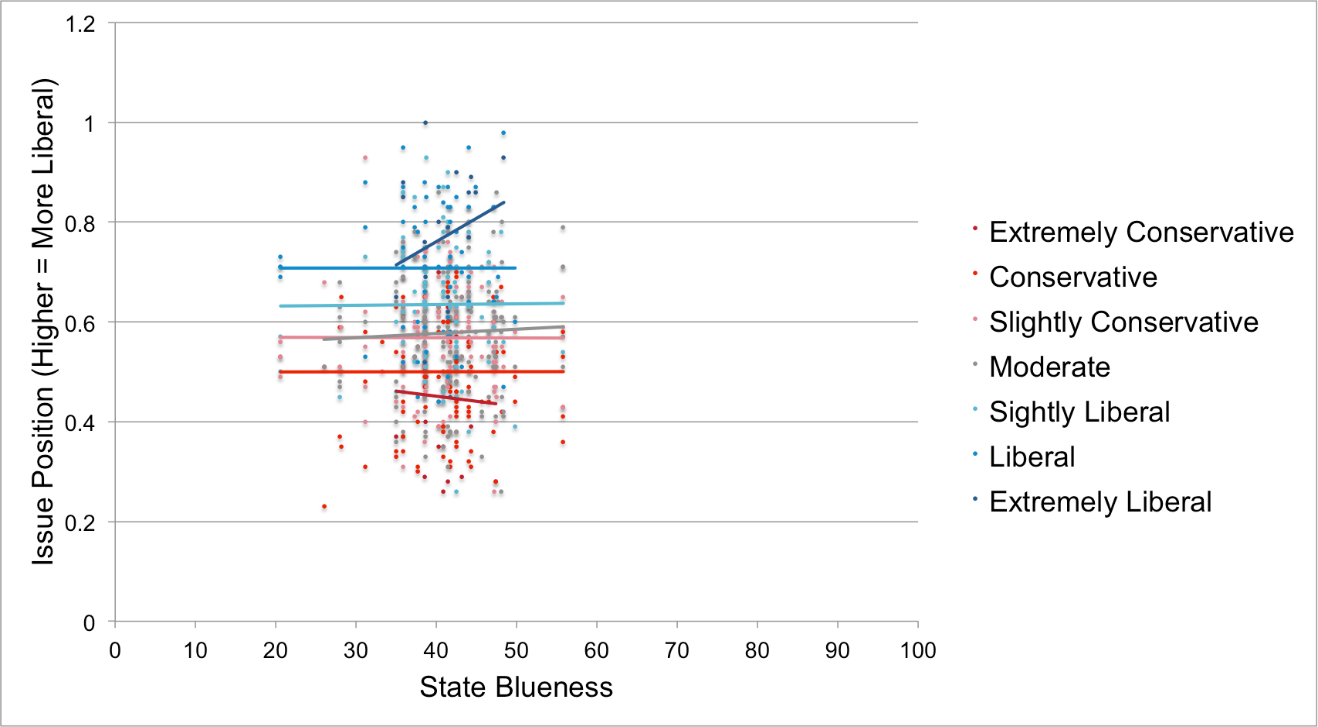
**

Figure Q


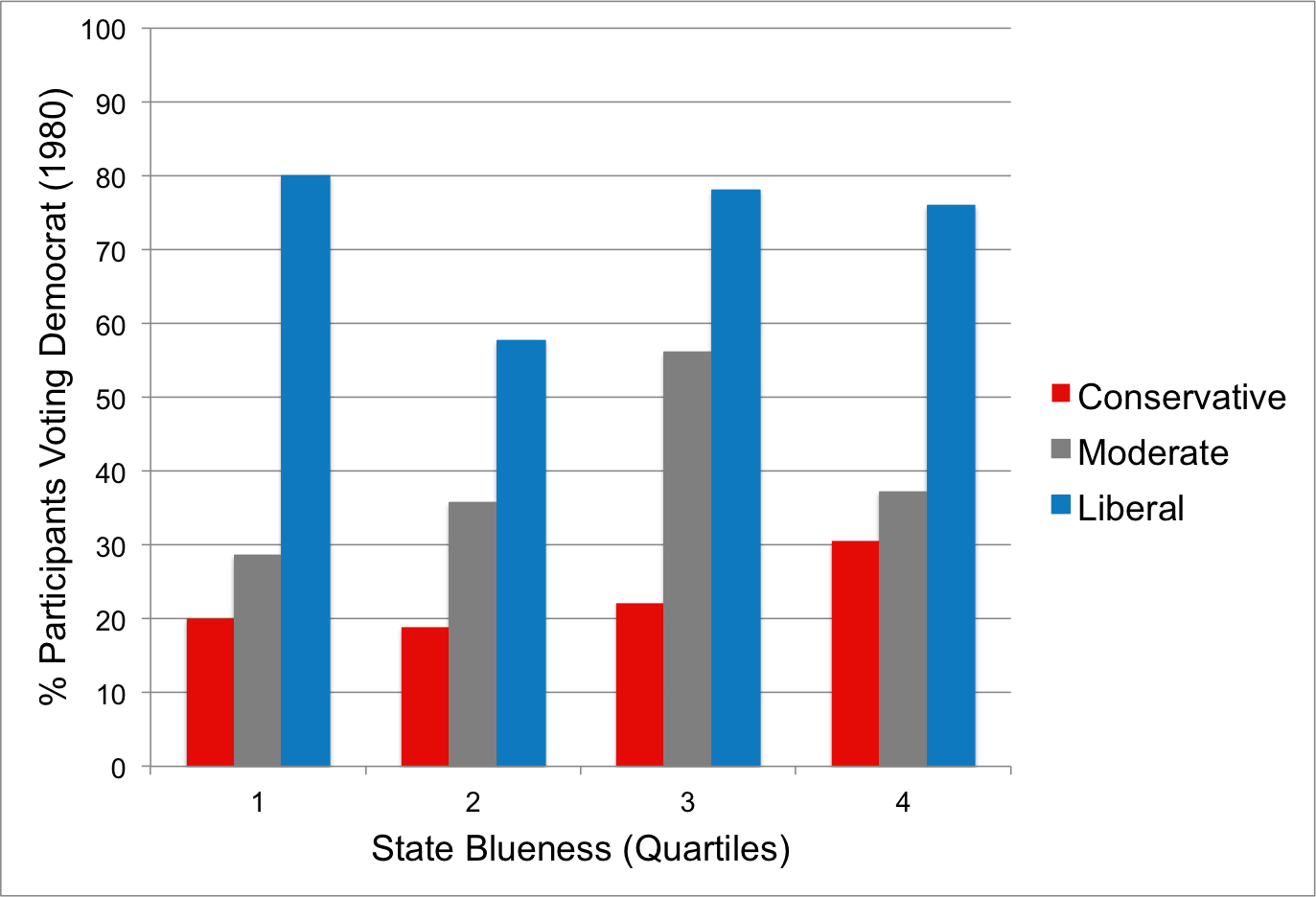


Figure R

**
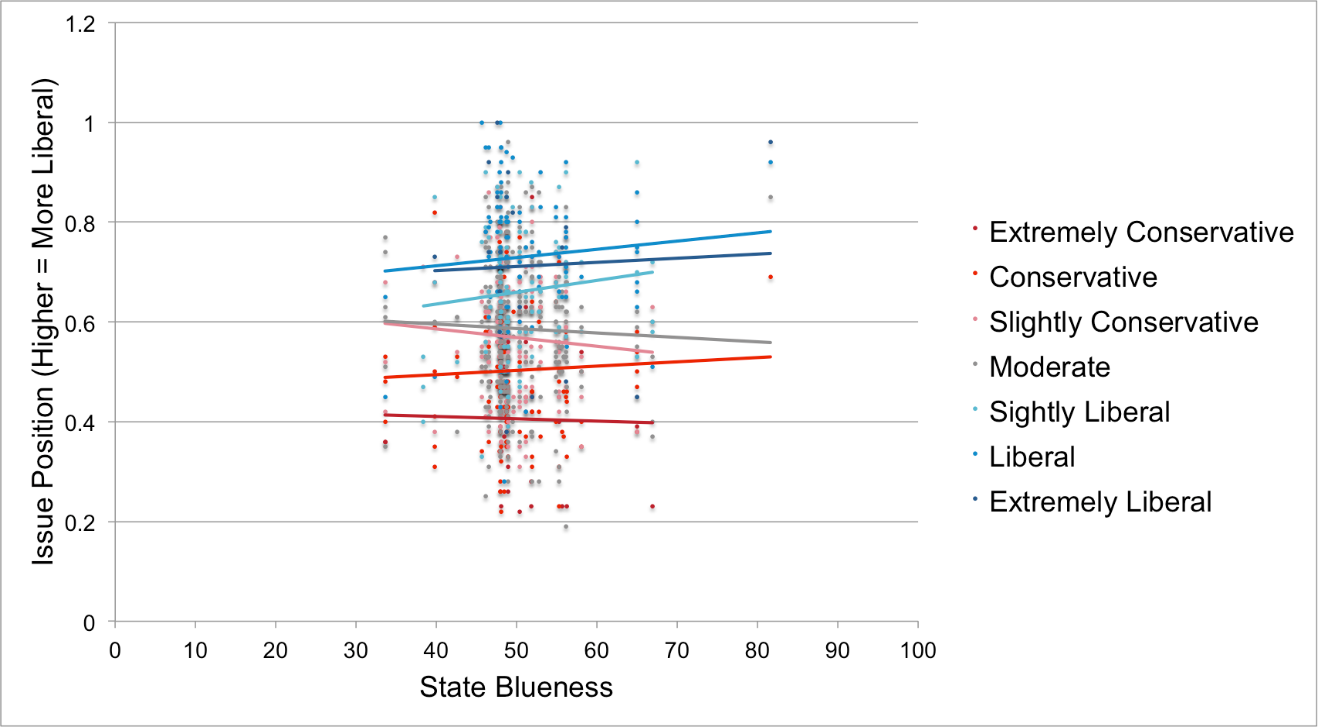
**

Figure S


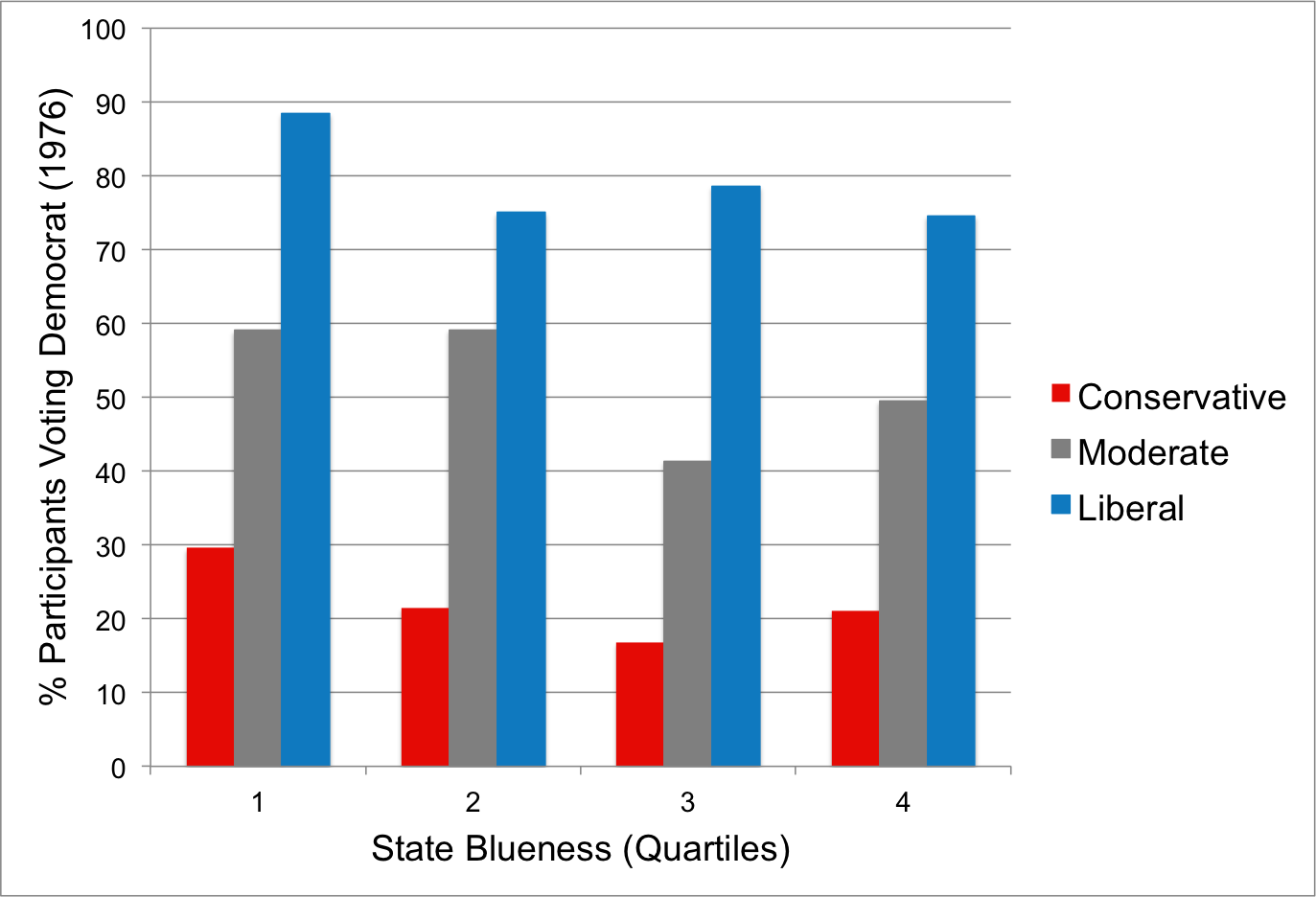


Figure T

**
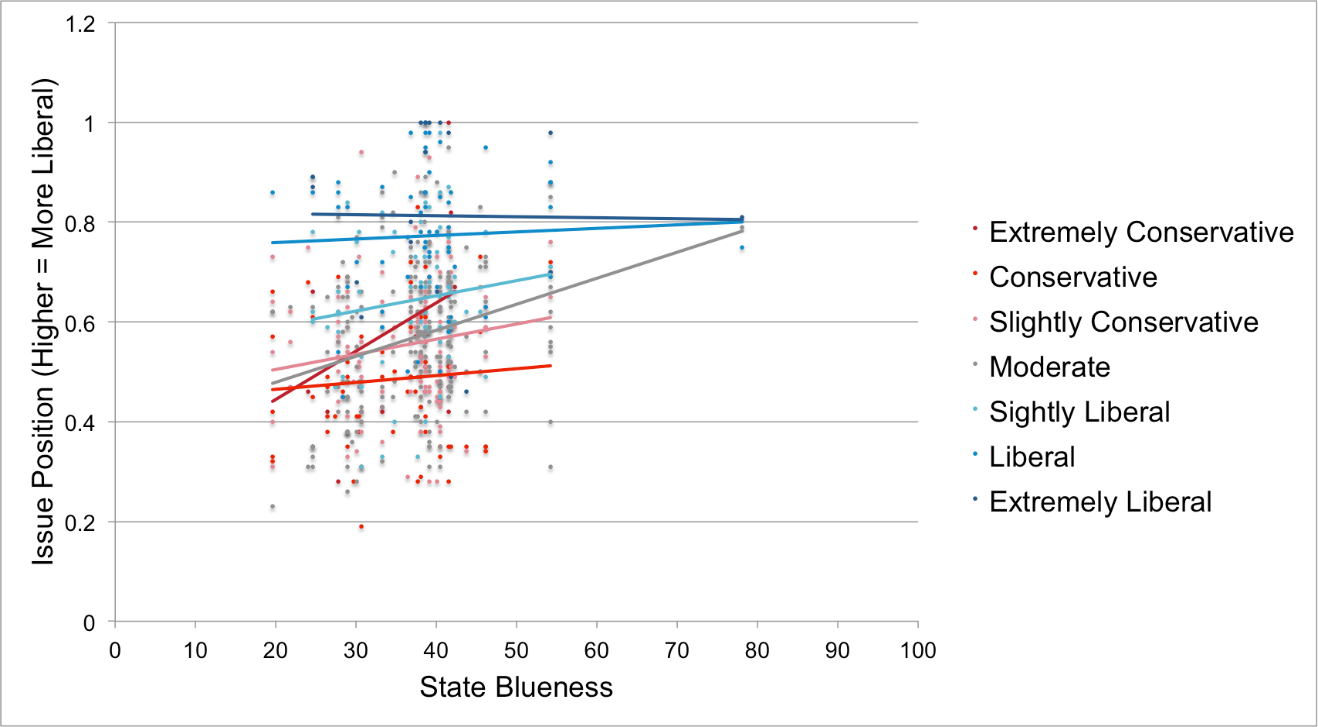
**

Figure U


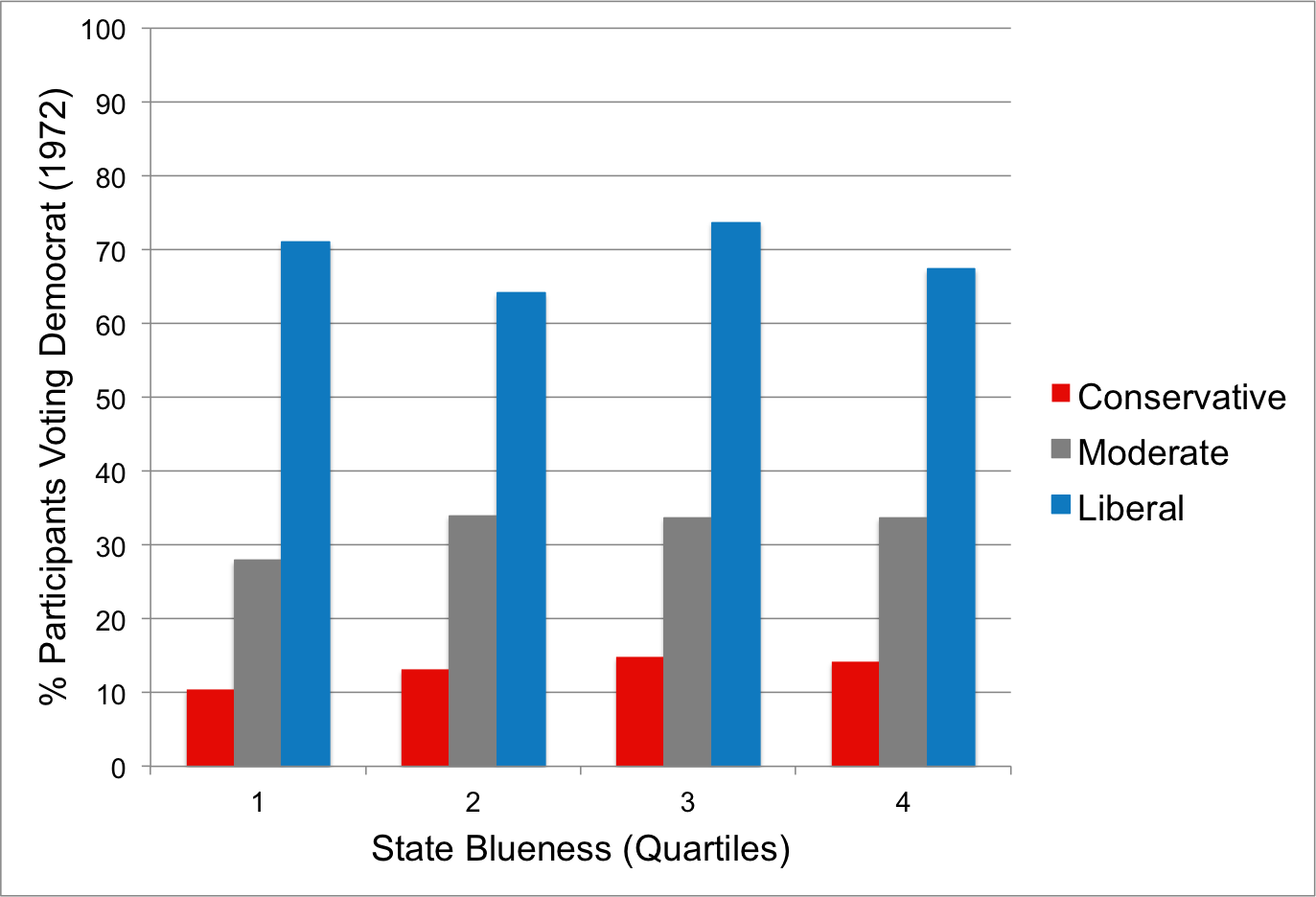


Figure V

**
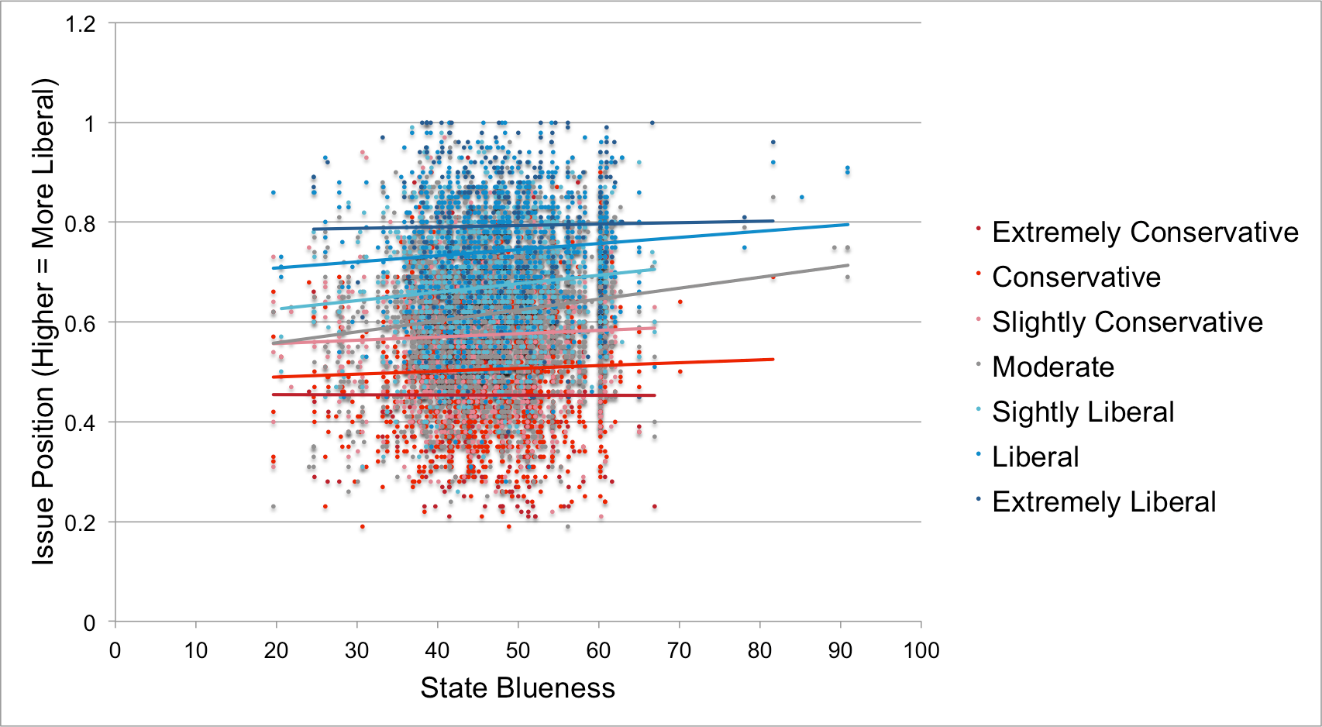
**

Figure W


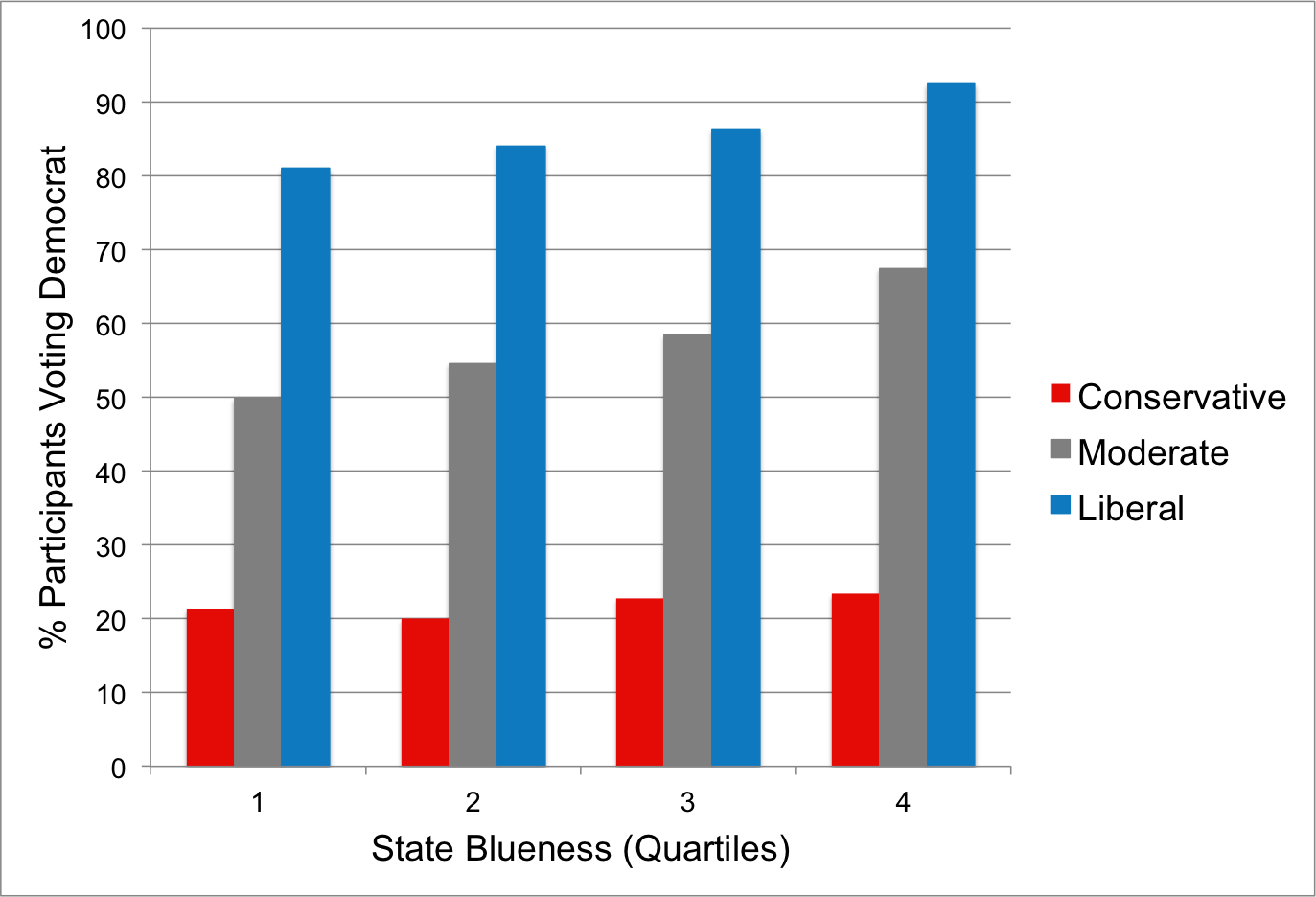


Figure X

Table A

| Political Identity | *N* | *M* | *SD* |
| --- | --- | --- | --- |
| 1. Extremely conservative | 81 | .41 | .13 |
| 2. Conservative | 373 | .48 | .12 |
| 3. Slightly conservative | 298 | .55 | .11 |
| 4. Moderate, middle of the road | 540 | .63 | .11 |
| 5. Slightly liberal | 223 | .67 | .10 |
| 6. Liberal | 228 | .75 | .11 |
| 7. Extremely liberal | 66 | .80 | .12 |

Table B

|  | *n* | *B* | *SE* | *t* | *p* |
| --- | --- | --- | --- | --- | --- |
| **Extremely conservative** | **81** | **.003** | **.001** | **4.18** | **<.001** |
| **Conservative** | **373** | **.002** | **.000** | **4.45** | **<.001** |
| **Slightly conservative** | **298** | **.002** | **.000** | **4.43** | **<.001** |
| **Moderate, middle of the road** | **540** | **.001** | **.000** | **3.09** | **.002** |
| Slightly liberal | 223 | .000 | .000 | .97 | .330 |
| Liberal | 228 | .000 | .001 | -.36 | .723 |
| Extremely liberal | 66 | -.001 | .000 | -1.07 | .285 |

**Table C**

| Issue | *R^2^_change_* | *F*(1, 1806) | *p* |
| --- | --- | --- | --- |
| Aggregate of all 9 issues | .004 | 12.32 | <.001 |
| 1. Government health insurance | .000 | .12 | .735 |
| 2. Guaranteed jobs and income | .000 | .67 | .412 |
| 3. Aid to Blacks | .001 | 1.22 | .271 |
| 4. Abortion | .006 | 13.49 | <.001 |
| 5. Government services spending | .000 | .48 | .488 |
| 6. Defense spending | .000 | .84 | .360 |
| 7. Affirmative action | .000 | .06 | .812 |
| 8. Protect homosexuals against discrimination | .006 | 11.53 | .001 |
| 9. Gays in the military | .009 | 18.02 | <.001 |

Table D

|  | Political Identity | | | | | | | Total |
| --- | --- | --- | --- | --- | --- | --- | --- | --- |
|  | 1 | 2 | 3 | 4 | 5 | 6 | 7 |  |
| Red States | 44 | 69 | 100 | 100 | 100 | 99 | 76 | 588 |
| Blue States | 77 | 98 | 99 | 102 | 100 | 100 | 105 | 681 |
| Total | 121 | 167 | 199 | 202 | 200 | 199 | 181 | 1269 |

**Table E**

| Political Identity | *N* | *M* | *SD* |
| --- | --- | --- | --- |
| 1. Strong conservative | 121 | -1.67 | 2.02 |
| 2. Conservative | 167 | -1.64 | 1.72 |
| 3. Moderate conservative | 199 | -.74 | 1.31 |
| 4. Moderate | 202 | .80 | 1.44 |
| 5. Moderate liberal | 200 | 1.81 | 1.45 |
| 6. Liberal | 199 | 2.80 | 1.24 |
| 7. Strong liberal | 181 | 3.34 | 1.48 |

Table F

|  | *B* | *SE* | *t* | *p* |
| --- | --- | --- | --- | --- |
| **Strong Conservatives** | **.031** | **.006** | **5.31** | **<.001** |
| **Conservatives** | **.025** | **.005** | **5.50** | **<.001** |
| **Moderate Conservatives** | **.019** | **.004** | **5.47** | **<.001** |
| **Moderates** | **.013** | **.003** | **4.55** | **<.001** |
| **Moderate Liberals** | **.007** | **.003** | **2.41** | **.016** |
| Liberals | .001 | .004 | .38 | .701 |
| Strong Liberals | -.004 | .005 | -.87 | .382 |

**Table G**

| Issue | *R^2^_change_* | *F*(1, 1266) | *p* |
| --- | --- | --- | --- |
| Aggregate of all 10 issues | .005 | 15.76 | <.001 |
| 1. A strong military | .003 | 4.54 | .033 |
| 2. Women’s reproductive rights | .002 | 3.64 | .057 |
| 3. Same sex marriage | .001 | 2.82 | .093 |
| 4. Capital punishment | .006 | 8.59 | .003 |
| 5. School prayer | .004 | 8.12 | .004 |
| 6. Social welfare | .002 | 4.13 | .042 |
| 7. Affordable care act | .007 | 13.83 | <.001 |
| 8. Off shore drilling | .001 | 1.91 | .168 |
| 9. Raising the minimum wage | .003 | 4.95 | .026 |
| 10. Enhanced interrogation techniques | .000 | .094 | .759 |

**Table H**

| Year | Alpha | Variable names | Variable labels |
| --- | --- | --- | --- |
| 1972 | .58 | VCF0806  VCF0809  VCF0814  VCF0830  VCF0834  VCF0837 | ISSUES: Government Health Insurance  ISSUES: Guaranteed Jobs and Income  ISSUES: Civil Rights Pushes Too Fast or Not Fast Enough  ISSUES: Aid to Blacks Scale  ISSUES: Women Equal Role Scale  ISSUES: When should abortion be allowed |
| 1976 | .57 | VCF0806  VCF0809  VCF0814  VCF0830  VCF0834  VCF0837 | ISSUES: Government Health Insurance  ISSUES: Guaranteed Jobs and Income  ISSUES: Civil Rights Pushes Too Fast or Not Fast Enough  ISSUES: Aid to Blacks Scale  ISSUES: Women Equal Role Scale  ISSUES: When should abortion be allowed |
| 1980 | .59 | VCF0809  VCF0814  VCF0830  VCF0834  VCF0838  VCF0843 | ISSUES: Guaranteed Jobs and Income  ISSUES: Civil Rights Pushes Too Fast or Not Fast Enough  ISSUES: Aid to Blacks Scale  ISSUES: Women Equal Role Scale  ISSUES: By Law, When Should Abortion Be Allowed  ISSUES: Defense Spending Scale |
| 1984 | .62 | VCF0806  VCF0809  VCF0814  VCF0830  VCF0834  VCF0838  VCF0839  VCF0843 | ISSUES: Government Health Insurance  ISSUES: Guaranteed Jobs and Income  ISSUES: Civil Rights Pushes Too Fast or Not Fast Enough  ISSUES: Aid to Blacks Scale  ISSUES: Women Equal Role Scale  ISSUES: By Law, When Should Abortion Be Allowed  ISSUES: Government Services-Spending Scale  ISSUES: Defense Spending Scale |
| 1988 | .66 | VCF0806  VCF0809  VCF0814  VCF0830  VCF0834  VCF0838  VCF0839  VCF0843  VCF0876a | ISSUES: Government Health Insurance  ISSUES: Guaranteed Jobs and Income  ISSUES: Civil Rights Pushes Too Fast or Not Fast Enough  ISSUES: Aid to Blacks Scale  ISSUES: Women Equal Role Scale  ISSUES: By Law, When Should Abortion Be Allowed  ISSUES: Government Services-Spending Scale  ISSUES: Defense Spending Scale  ISSUES: Strength of Position on Law to Protect Homosexuals |
| 1992 | .75 | VCF0806  VCF0809  VCF0814  VCF0830  VCF0834  VCF0838  VCF0839  VCF0843  VCF0867a  VCF0876a  VCF0877a | ISSUES: Government Health Insurance  ISSUES: Guaranteed Jobs and Income  ISSUES: Civil Rights Pushes Too Fast or Not Fast Enough  ISSUES: Aid to Blacks Scale  ISSUES: Women Equal Role Scale  ISSUES: By Law, When Should Abortion Be Allowed  ISSUES: Government Services-Spending Scale  ISSUES: Defense Spending Scale  ISSUES: Affirmative Action in Hiring/Promotion [2 of 2]  ISSUES: Strength of Position on Law to Protect Homosexuals  ISSUES: Strength of Position on Gays in the Military |
| 1996 | .76 | VCF0806  VCF0809  VCF0830  VCF0834  VCF0838  VCF0839  VCF0843  VCF0867a  VCF0876a  VCF0877a | ISSUES: Government Health Insurance  ISSUES: Guaranteed Jobs and Income  ISSUES: Aid to Blacks Scale  ISSUES: Women Equal Role Scale  ISSUES: By Law, When Should Abortion Be Allowed  ISSUES: Government Services-Spending Scale  ISSUES: Defense Spending Scale  ISSUES: Affirmative Action in Hiring/Promotion [2 of 2]  ISSUES: Strength of Position on Law to Protect Homosexuals  ISSUES: Strength of Position on Gays in the Military |
| 2000 | .70 | VCF0806  VCF0809  VCF0830  VCF0834  VCF0838  VCF0839  VCF0843  VCF0867a  VCF0876a  VCF0877a | ISSUES: Government Health Insurance  ISSUES: Guaranteed Jobs and Income  ISSUES: Aid to Blacks Scale  ISSUES: Women Equal Role Scale  ISSUES: By Law, When Should Abortion Be Allowed  ISSUES: Government Services-Spending Scale  ISSUES: Defense Spending Scale  ISSUES: Affirmative Action in Hiring/Promotion [2 of 2]  ISSUES: Strength of Position on Law to Protect Homosexuals  ISSUES: Strength of Position on Gays in the Military |
| 2004 | .76 | VCF0806  VCF0809  VCF0830  VCF0834  VCF0838  VCF0839  VCF0843  VCF0867a  VCF0876a  VCF0877a | ISSUES: Government Health Insurance  ISSUES: Guaranteed Jobs and Income  ISSUES: Aid to Blacks Scale  ISSUES: Women Equal Role Scale  ISSUES: By Law, When Should Abortion Be Allowed  ISSUES: Government Services-Spending Scale  ISSUES: Defense Spending Scale  ISSUES: Affirmative Action in Hiring/Promotion [2 of 2]  ISSUES: Strength of Position on Law to Protect Homosexuals  ISSUES: Strength of Position on Gays in the Military |
| 2008 | .71 | VCF0806  VCF0809  VCF0830  VCF0834  VCF0838  VCF0839  VCF0843  VCF0867a  VCF0876a  VCF0877a | ISSUES: Government Health Insurance  ISSUES: Guaranteed Jobs and Income  ISSUES: Aid to Blacks Scale  ISSUES: Women Equal Role Scale  ISSUES: By Law, When Should Abortion Be Allowed  ISSUES: Government Services-Spending Scale  ISSUES: Defense Spending Scale  ISSUES: Affirmative Action in Hiring/Promotion [2 of 2]  ISSUES: Strength of Position on Law to Protect Homosexuals  ISSUES: Strength of Position on Gays in the Military |
| 2012 | .79 | VCF0806  VCF0809  VCF0830  VCF0838  VCF0839  VCF0843  VCF0867a  VCF0876a  VCF0877a | ISSUES: Government Health Insurance  ISSUES: Guaranteed Jobs and Income  ISSUES: Aid to Blacks Scale  ISSUES: By Law, When Should Abortion Be Allowed  ISSUES: Government Services-Spending Scale  ISSUES: Defense Spending Scale  ISSUES: Affirmative Action in Hiring/Promotion [2 of 2]  ISSUES: Strength of Position on Law to Protect Homosexuals  ISSUES: Strength of Position on Gays in the Military |

**Table I**

|  | *n* | *B* | *SE* | *t* | *p* |
| --- | --- | --- | --- | --- | --- |
| Extremely conservative | 29 | .001 | .002 | .65 | .52 |
| Conservative | 88 | .002 | .001 | 1.50 | .134 |
| **Slightly conservative** | **75** | **.002** | **.001** | **2.90** | **.004** |
| **Moderate, middle of the road** | **137** | **.003** | **.001** | **4.44** | **<.001** |
| **Slightly liberal** | **53** | **.004** | **.001** | **4.68** | **<.001** |
| **Liberal** | **66** | **.005** | **.001** | **4.11** | **<.001** |
| **Extremely liberal** | **25** | **.005** | **.001** | **3.60** | **<.001** |

Table J

|  | *n* | *B* | *SE* | *p* |
| --- | --- | --- | --- | --- |
| Conservative | 443 | .010 | .017 | .545 |
| Moderate | 326 | .030 | .018 | .097 |
| Liberal | 372 | .040 | .026 | .119 |

**Table K**

|  | *n* | *B* | *SE* | *t* | *p* |
| --- | --- | --- | --- | --- | --- |
| Extremely conservative | 21 | .001 | .001 | .39 | .694 |
| Conservative | 134 | .000 | .001 | .38 | .707 |
| Slightly conservative | 102 | .000 | .001 | .30 | .767 |
| Moderate, middle of the road | 167 | .000 | .001 | .08 | .939 |
| Slightly liberal | 55 | .000 | .001 | -.13 | .894 |
| Liberal | 66 | .000 | .001 | -.23 | .816 |
| Extremely liberal | 15 | .000 | .001 | -.28 | .781 |

Table L

|  | *n* | *B* | *SE* | *p* |
| --- | --- | --- | --- | --- |
| Conservative | 300 | -.027 | .024 | .263 |
| Moderate | 190 | .003 | .023 | .896 |
| Liberal | 170 | .060 | .033 | .068 |

Table M

|  | *n* | *B* | *SE* | *t* | *p* |
| --- | --- | --- | --- | --- | --- |
| Extremely conservative | 8 | .004 | .002 | 1.75 | .082 |
| Conservative | 39 | .003 | .002 | 1.90 | .058 |
| **Slightly conservative** | **37** | **.002** | **.001** | **2.01** | **.046** |
| Moderate, middle of the road | 72 | .002 | .001 | 1.62 | .107 |
| Slightly liberal | 28 | .001 | .001 | .74 | .458 |
| Liberal | 17 | .000 | .002 | .12 | .904 |
| Extremely liberal | 7 | .000 | .002 | -.23 | .822 |

Table N

|  | *n* | *B* | *SE* | *p* |
| --- | --- | --- | --- | --- |
| Conservative | 196 | .060 | .038 | .121 |
| Moderate | 140 | .047 | .029 | .106 |
| Liberal | 119 | .042 | .049 | .392 |

Table O

|  | *n* | *B* | *SE* | *t* | *p* |
| --- | --- | --- | --- | --- | --- |
| Extremely conservative | 26 | .001 | .001 | .81 | .418 |
| Conservative | 186 | .001 | .001 | 1.32 | .189 |
| Slightly conservative | 161 | .001 | .001 | 1.94 | .052 |
| **Moderate, middle of the road** | **221** | **.002** | **.001** | **2.13** | **.034** |
| Slightly liberal | 121 | .002 | .001 | 1.84 | .066 |
| Liberal | 86 | .002 | .001 | 1.54 | .124 |
| Extremely liberal | 14 | .002 | .002 | 1.33 | .185 |

Table P

|  | *n* | *B* | *SE* | *p* |
| --- | --- | --- | --- | --- |
| Conservative | 417 | .010 | .026 | .691 |
| Moderate | 219 | .026 | .033 | .423 |
| Liberal | 222 | .000 | .064 | .999 |

Table Q

|  | *n* | *B* | *SE* | *t* | *p* |
| --- | --- | --- | --- | --- | --- |
| Extremely conservative | 29 | .002 | .002 | .91 | .365 |
| Conservative | 176 | .002 | .001 | 1.38 | .167 |
| **Slightly conservative** | **224** | **.002** | **.001** | **2.13** | **.033** |
| **Moderate, middle of the road** | **318** | **.002** | **.001** | **2.52** | **.012** |
| **Slightly liberal** | **159** | **.002** | **.001** | **2.01** | **.045** |
| Liberal | 135 | .002 | .002 | 1.52 | .129 |
| Extremely liberal | 28 | .003 | .002 | 1.22 | .225 |

Table R

|  | *n* | *B* | *SE* | *p* |
| --- | --- | --- | --- | --- |
| Conservative | 450 | .016 | .022 | .464 |
| Moderate | 309 | .035 | .025 | .163 |
| Liberal | 317 | .044 | .042 | .298 |

Table S

|  | *n* | *B* | *SE* | *t* | *p* |
| --- | --- | --- | --- | --- | --- |
| Extremely conservative | 31 | .003 | .002 | 1.52 | .129 |
| **Conservative** | **157** | **.003** | **.002** | **2.11** | **.035** |
| **Slightly conservative** | **187** | **.003** | **.001** | **3.00** | **.003** |
| **Moderate, middle of the road** | **220** | **.003** | **.001** | **3.39** | **.001** |
| **Slightly liberal** | **108** | **.003** | **.001** | **2.61** | **.009** |
| Liberal | 66 | .003 | .002 | 1.88 | .061 |
| Extremely liberal | 22 | .003 | .002 | 1.42 | .155 |

Table T

|  | *n* | *B* | *SE* | *p* |
| --- | --- | --- | --- | --- |
| Conservative | 455 | -.006 | .026 | .814 |
| Moderate | 275 | -.004 | .028 | .880 |
| **Liberal** | **205** | **.089** | **.040** | **.027** |

Table U

|  | *n* | *B* | *SE* | *t* | *p* |
| --- | --- | --- | --- | --- | --- |
| Extremely conservative | 7 | .000 | .003 | .02 | .987 |
| Conservative | 89 | .000 | .002 | .13 | .899 |
| Slightly conservative | 105 | .000 | .001 | .34 | .736 |
| Moderate, middle of the road | 161 | .001 | .001 | .52 | .604 |
| Slightly liberal | 65 | .001 | .002 | .48 | .630 |
| Liberal | 57 | .001 | .002 | .42 | .678 |
| Extremely liberal | 5 | .001 | .001 | .22 | .829 |

Table V

|  | *n* | *B* | *SE* | *p* |
| --- | --- | --- | --- | --- |
| **Conservative** | **462** | **.073** | **.023** | **.002** |
| Moderate | 320 | .025 | .020 | .214 |
| Liberal | 279 | -.034 | .025 | .174 |

Table W

|  | *n* | *B* | *SE* | *t* | *p* |
| --- | --- | --- | --- | --- | --- |
| Extremely conservative | 19 | .000 | .002 | -.18 | .859 |
| Conservative | 131 | .000 | .001 | -.12 | .783 |
| Slightly conservative | 142 | .000 | .001 | .01 | .992 |
| Moderate, middle of the road | 178 | .000 | .001 | .21 | .836 |
| Slightly liberal | 92 | .000 | .001 | .30 | .766 |
| Liberal | 66 | .001 | .002 | .31 | .755 |
| Extremely liberal | 19 | .001 | .002 | .31 | .756 |

Table X

|  | *n* | *B* | *SE* | *p* |
| --- | --- | --- | --- | --- |
| Conservative | 290 | .035 | .024 | .147 |
| Moderate | 175 | .038 | .031 | .224 |
| Liberal | 137 | .031 | .047 | .515 |

Table Y

|  | *n* | *B* | *SE* | *t* | *p* |
| --- | --- | --- | --- | --- | --- |
| Extremely conservative | 34 | -.001 | .002 | -.59 | .556 |
| Conservative | 154 | -.001 | .001 | -.47 | .640 |
| Slightly conservative | 166 | .000 | .001 | -.21 | .836 |
| Moderate, middle of the road | 312 | .000 | .001 | .25 | .802 |
| Slightly liberal | 109 | .001 | .001 | .63 | .532 |
| Liberal | 97 | .001 | .001 | .77 | .443 |
| Extremely liberal | 20 | .001 | .001 | .80 | .425 |

Table Z

|  | *n* | *B* | *SE* | *p* |
| --- | --- | --- | --- | --- |
| Conservative | 417 | -.012 | .017 | .467 |
| Moderate | 352 | -.005 | .016 | .743 |
| Liberal | 231 | -.007 | .091 | .763 |

Table AA

|  | *n* | *B* | *SE* | *t* | *p* |
| --- | --- | --- | --- | --- | --- |
| Extremely conservative | 12 | .003 | .002 | 1.87 | .062 |
| **Conservative** | **82** | **.003** | **.001** | **2.43** | **.016** |
| **Slightly conservative** | **126** | **.003** | **.001** | **3.26** | **.001** |
| **Moderate, middle of the road** | **214** | **.003** | **.001** | **3.90** | **<.001** |
| **Slightly liberal** | **70** | **.003** | **.001** | **3.29** | **.001** |
| **Liberal** | **62** | **.003** | **.001** | **2.35** | **.019** |
| Extremely liberal | 18 | .003 | .002 | 1.71 | .088 |

Table BB

|  | *n* | *B* | *SE* | *p* |
| --- | --- | --- | --- | --- |
| Conservative | 453 | .033 | .017 | .059 |
| Moderate | 421 | .018 | .012 | .132 |
| Liberal | 318 | .008 | .013 | .532 |

Table CC

|  | *n* | *B* | *SE* | *t* | *p* |
| --- | --- | --- | --- | --- | --- |
| Extremely conservative | 297 | .001 | .000 | 1.74 | .082 |
| **Conservative** | **1609** | **.001** | **.000** | **3.21** | **.001** |
| **Slightly conservative** | **1623** | **.001** | **.000** | **5.62** | **<.001** |
| **Moderate, middle of the road** | **2540** | **.001** | **.000** | **7.74** | **<.001** |
| **Slightly liberal** | **1082** | **.002** | **.000** | **7.18** | **<.001** |
| **Liberal** | **946** | **.002** | **.000** | **5.92** | **<.001** |
| **Extremely liberal** | **239** | **.002** | **.000** | **5.02** | **<.001** |

Table DD

|  | *n* | *B* | *SE* | *p* |
| --- | --- | --- | --- | --- |
| **Conservative** | **5395** | **.010** | **.004** | **.014** |
| **Moderate** | **3943** | **.036** | **.004** | **<.001** |
| **Liberal** | **3504** | **.045** | **.006** | **<.001** |

Table EE

|  | Step 1 (political identity) | | Step 2 (state blueness) | | Step 3 (interaction) | |
| --- | --- | --- | --- | --- | --- | --- |
| Year | *R^2^* | *p* | *R^2^_change_* | *p* | *R^2^_change_* | *p* |
| 1972 | .228** | <.001 | .020** | <.001 | .000 | .890 |
| 1976 | .240** | <.001 | .000 | .818 | .001 | .425 |
| 1980 | .247** | <.001 | .000 | .882 | .000 | .783 |
| 1984 | .201** | <.001 | .000 | .634 | .000 | .829 |
| 1988 | .230** | <.001 | .011** | <.001 | .000 | .956 |
| 1992 | .286** | <.001 | .004* | .012 | .000 | .786 |
| 1996 | .406** | <.001 | .003* | .037 | .000 | .619 |
| 2000 | .374** | <.001 | .009^†^ | .093 | .004 | .278 |
| 2004 | .415** | <.001 | .000 | .885 | .000 | .713 |
| 2008 | .352** | <.001 | .026** | <.001 | .004^†^ | .096 |
| 2012 | .442** | <.001 | .004** | <.001 | .002** | .004 |
| Combined | .317** | <.001 | .005** | <.001 | .000* | .046 |

Note: Key analysis is Step 2. ^†^*p* < .10, **p* < .05, ***p* < .01.

Table FF

|  | Step 1 (political identity) | | Step 2 (state blueness) | | Step 3 (interaction) | |
| --- | --- | --- | --- | --- | --- | --- |
| Year | χ^2^ | *p* | χ^2^ | *p* | χ^2^ | *p* |
| 1972 | 266.15** | <.001 | 5.14* | .023 | 1.30 | .522 |
| 1976 | 211.48** | <.001 | .63 | .426 | .10 | .952 |
| 1980 | 104.05** | <.001 | 4.03* | .045 | .02 | .991 |
| 1984 | 220.40** | <.001 | 3.79^†^ | .052 | 10.07** | .007 |
| 1988 | 219.26** | <.001 | .46 | .498 | 4.49 | .102 |
| 1992 | 355.93** | <.001 | 3.09^†^ | .079 | .49 | .782 |
| 1996 | 343.28** | <.001 | .59 | .442 | .21 | .901 |
| 2000 | 140.17** | <.001 | 6.00* | .014 | .09 | .952 |
| 2004 | 280.12** | <.001 | .03 | .875 | 4.32 | .115 |
| 2008 | 370.82** | <.001 | 4.50* | .034 | 1.19 | .552 |
| 2012 | 1670.66** | <.001 | 9.84** | .002 | 2.31 | .315 |
| Combined | 3991.61** | <.001 | 117.30** | <.001 | 32.29** | <.001 |

Note: Key analysis is Step 2. ^†^*p* < .10, **p* < .05, ***p* < .01

**References**

Oppenheimer DM, Meyvis T, Davidenko N. Instructional manipulation checks: Detecting satisficing to increase statistical power. J Exp Soc Psychol. 2009;45: 867-872.

Vallone RP, Ross L, Lepper MR. The hostile media phenomenon: biased perception and perceptions of media bias in coverage of the Beirut massacre. J Pers Soc Psychol. 1985;49: 577.
